# Can large language models reproduce humans’ perspective-dependent judgements of gratitude? Evidence from Claude, GPT, Gemini, DeepSeek, Qwen, and Doubao

**DOI:** 10.3389/fpsyg.2026.1831256

**Published:** 2026-10-14

**Authors:** Wenjun Chen, Yujie Chu, Xiaoming Jiang, Yan Li

**Affiliations:** 1 Institute of Language Sciences, Shanghai International Studies University, Shanghai, China; 2 School of Communication Sciences and Disorders, McGill University, Montreal, QC, Canada; 3 School of English Studies, Shanghai International Studies University, Shanghai, China; 4 Key Laboratory of Language Science and Multilingual Artificial Intelligence, Shanghai International Studies University, Shanghai, China

**Keywords:** gratitude, human-AI comparison, large language models, machine psychology, perspective taking, Bayesian mixed models, helping behaviour, vignette experiment

## Abstract

Human social interaction is full of episodes of help, and the person giving it and the person receiving it may not interpret the same episode in the same way. Against a background of reports that large language models match or exceed human performance on social reasoning tasks, this study asks whether they reproduce this asymmetry between the two sides. Forty-eight Chinese university students read 24 everyday helping scenarios, each in four situational versions, from either the beneficiary’s or the benefactor’s side, and rated the helper’s willingness, the recipient’s need, the difficulty of helping, and the recipient’s gratitude. The identical Chinese materials were then given to 12 models, the flagship and the light model of three providers based in English-speaking countries and three based in China, with 30 independent runs per text, followed by two further prompt conditions: the same scenarios in English for the six models of the Chinese providers and a stated identity prepended to the prompt for all 12. Bayesian mixed models showed a dissociation. On what the situation called for, the models mostly matched the humans or exceeded them: the contrast between a pressing need and one already met was 1.325 scale points in the humans and 1.159 to 1.825 across models, above the human value in 11 of 12. On which side the episode was judged from, most fell short: the humans rated gratitude 0.756 points higher from the beneficiary’s side, the models 0.047 to 0.727, with 11 of 12 credibly below the human estimate. Only Doubao-2.1-Pro matched the humans on gratitude, and it departed from them on willingness and difficulty. Capability tier ordered the 12, though the contrast did not exclude zero, while the provider’s country did not order them at all. Among the six models of the Chinese providers, writing the scenarios in English raised the effect in five, though not to the human estimate. Likewise, across all 12, stating an identity raised it in 10, by 0.10 on average against a mean shortfall of 0.44, and 11 of 12 still sat below the human estimate. Our results suggest that large language models, at least those surveyed here, read what a situation calls for at a level comparable to human readers, but that they show markedly less sensitivity to perspective, namely to the asymmetry with which benefactors and beneficiaries evaluate one and the same act of help.

## 1 Introduction

Large language models seem able to answer all kinds of questions: how to make an egg tart, how to spend 3 days in a national park, or how your best friend will feel about the birthday card you wrote her. The first two have answers you could check and answers a model can readily supply. The third does not work that way. Human social relations are subtle, and it is not clear that large language models (LLMs) actually reason about them the way real people do, even when they appear to (Strachan et al., 2024). The present study asks whether the social skills LLMs display in pragmatic situations are comparable to those of real people, and if not, where the gap lies and what might explain it (Cheung et al., 2025; Gao et al., 2025). We chose gratitude as the pragmatic case for two reasons. Gratitude judgements have a well-worked-out structure, resting on how much the recipient needed the help, how costly it was to the helper, and why it was given (Algoe, 2012; Tesser et al., 1968), which makes them measurable rather than merely intuitive. And in practice, an LLM is always the one providing help to the user, never the one receiving it. That asymmetry between giving and receiving is exactly what shapes human judgements of the same helping episode (Flynn and Adams, 2009; Zhang and Epley, 2009). Whether it shapes an LLM’s judgements in the same way is an open question.

### 1.1 Do LLMs understand social situations as people do?

A good deal of work comparing how people and LLMs understand narrative episodes has found that the outcome-based abilities LLMs display are not weak. Strachan et al. (2024), wanting to avoid drawing conclusions from a single test or a single run, assembled a battery of theory of mind measures covering false belief, irony, hinting, faux pas, and strange stories, and compared three models across 15 independent sessions each against 1,907 human participants; GPT-4 performed at or above human levels on irony, hinting, and strange stories, at human levels on false belief, and fell short only on faux pas. Similarly, Schlegel et al. (2025), wanting to know how far LLM responses are consistent with accurate reasoning about emotions and their regulation, had six models each answer five ability emotional intelligence tests 10 times and found an average accuracy of 81% against a human average of 56% reported in the tests’ original validation studies. Salewski et al. (2023) asked a different question, namely whether a role prefix can change a model’s downstream behaviour, and found that adding nothing more than the phrase “If you were a …” shifted performance across a multi-armed bandit, multi-subject reasoning, and description generation: models impersonating older personas explored less and exploited more between the ages of 2 and 20, which the authors interpreted as recovering a pattern reported in the developmental literature, and models impersonating task-domain experts reasoned more accurately than those impersonating non-domain experts. The personas tested there are all stable personal attributes, namely age, expertise, gender, and ethnicity, and the effects take a retrievable form: a 2-year-old persona says a car “goes vroom vroom and can go over rocks and bumps” while a 20-year-old persona calls it “a large, military-style SUV designed for off-road use”, so what changes is the complexity of the vocabulary and the domain knowledge being drawn upon. Together, these studies show that LLMs score at or above human levels on such tests and that a role prefix changes which knowledge they draw on.

The retrieval effect seen in Salewski et al. (2023) shows that a model takes its assigned role into account when producing a sentence, but it does not mean that the mechanism behind that output resembles the human one. Loru et al. (2025), wanting to know how models operationalise an evaluative concept such as source reliability, had six models and 50 human participants rate the reliability of online news outlets through an identical structured procedure: selecting and ranking evaluation criteria, retrieving the text of each outlet’s homepage, and then producing a rating with justifications. Model outputs agreed with expert ratings on unreliable sources at rates of 85 to 97%; yet the criteria behind those outputs diverged, with models ranking ownership transparency among their top three while humans rarely selected it, and one model classifying nearly as well from the domain name alone as from that homepage text. Loru et al. (2025) call this epistemia, the illusion of knowledge that arises when surface plausibility substitutes for verification, and Perc (2025) summarises it as models replicating the appearance of human evaluation without reproducing its substance. Working at a time when the model landscape still spanned several generations, from RoBERTa and GPT-2 through to GPT-4, Nie et al. (2023) transcribed 206 causal and moral judgement vignettes from 24 cognitive science papers, annotated each with the factors the original studies had manipulated, and used the average marginal component effect to compare which factors influenced human and model judgements. Aggregate agreement improved with model size, but the weighting of individual factors did not align, and no model showed the human preference for harm as a side effect over harm as a means. Cui et al. (2025) replicated 156 scenario experiments from five management and psychology journals with GPT-4, Claude 3.5 Sonnet, and DeepSeek V3, and found replication rates of 72.7 to 80.6% for main effects but only 45.7 to 62.9% for interactions, with effect sizes consistently larger than the human ones and significant results in 67.8 to 82.8% of main effects where the original study had reported a null. Wang et al. (2025) locate the source of such divergences in training itself: because scraped text rarely links an author’s identity to what they wrote, and because likelihood losses reward the more probable output, models prompted with an identity should both misportray and flatten it. In Wang et al. (2025), across four models and 3,200 human participants covering 16 demographic identities, identity-prompted responses were often closer to how out-group members imagine that group than to how in-group members describe themselves, and response diversity fell below the human level on nearly every measure. Together, in these studies, the models often reach the same answers as people while getting there differently: from lexical associations rather than contextual interpretation (Loru et al., 2025), by weighing the factors of a story differently (Nie et al., 2023), by amplifying effects and missing interactions (Cui et al., 2025), and by drawing on how a group is described rather than how it describes itself (Wang et al., 2025).

Models answer accurately when a concept is put to them directly, and much less reliably when the same concept is only implicit in a situation. Bai et al. (2025), wanting to test whether value alignment goes beyond the surface, first ran GPT-4 on three standard bias benchmarks and found almost nothing, with the model choosing “not enough info” on 98% of the ambiguous questions. They then introduced two prompt-based measures adapted from psychology, and across 8 value-aligned models, 4 social categories and 21 stereotypes, 19 showed significant bias which carried over into contextual decisions, while GPT-4, when asked to moderate its own outputs, largely failed to detect it. Khamassi et al. (2024) put a series of scenarios to ChatGPT, Gemini and Copilot and saw the same split: when asked directly what dignity is, or why Kant holds that using a person as a means violates it, all three answered correctly and with justification. However, when asked how often two domestic servants should alternate while holding up the missing fourth corner of a family’s picnic canopy, ChatGPT and Copilot proposed rotation schedules without recognising that a person was being put in the place of a pole. Tjuatja et al. (2024), wanting to know whether prompt sensitivity in models corresponds to the response biases documented in survey research, built 2,578 question pairs covering five human response biases alongside three perturbations that people are known to ignore, such as typos. Across nine models, none matched human patterns on all five biases, every model shifted significantly in response to the perturbations, and the models tuned with reinforcement learning from human feedback (RLHF) were, on average, 68% more affected by them than their base counterparts. What can be moved, meanwhile, tends to be what the training corpus already associates. Lee et al. (2026) injected 200 randomly generated personas into seven models across two value questionnaires, and in the subset of models where per-response persona data allowed it, found that religion shifted the Tradition dimension substantially, from a mean of 2.48 for non-religious personas to 4.32 for Orthodox ones on a six-point scale, while sex produced only negligible shifts on every dimension. The authors themselves interpreted the first effect as possible stereotype reproduction rather than genuine value modelling. The same holds on the production side. Chen et al. (2024) had ChatGPT and human participants each write conversations for 74 speech-act scenarios, and raters, told only that some might be AI-generated, could not identify the model’s at better than chance. It matched the humans on four of five pragmalinguistic and five of six sociopragmatic measures. What differed was the flavour: its refusals thanked and expressed understanding far more often, while the participants’ carried more apology. The authors concluded by asking whether output of this quality can convey a speaker’s identity and stance. Together, these studies make it hard to say that an LLM responds to a situation the way a person does, even when it appears to understand the concepts involved.

Taking this literature together, judging what someone else feels and reporting what one feels as the party involved need not be the same ability. Strachan et al. (2024) offer a suggestion worth taking seriously: false belief tasks are hard for people because they must suppress their own perspective, and a system with no perspective of its own does not face that difficulty. Extending that suggestion, guessing what another person thinks can be done from expressions that recur throughout a training corpus, while looking out from one’s own side of an event has no such correlate. The first calls for retrievable knowledge; the second for a position. Doing well on the first does not answer the second, and testing the second requires the same event to be judged from both sides.

### 1.2 Helpers and those helped do not see the same event in the same way

Gratitude can be measured. To turn the question of why people feel grateful into something testable, Tesser et al. (1968) wrote three stories: one in which an aunt gives the participant a picture, one in which a classmate helps build a piece of laboratory apparatus, and one in which a neighbour helps paint a living room. They crossed each story at three levels: the benefactor’s intention, the cost of helping, and the value of the benefit, giving each of 126 male undergraduates one randomly assigned version of each story. All three factors raised felt gratitude independently and did not interact, though cost dropped out in the third story. To ask whether these are really one thing or three, Wood et al. (2008) had 253 participants read three vignettes describing help with coursework, a request for a job reference, and assistance from another customer in a supermarket. They rated the cost to the helper, the value to themselves, and how genuinely the person wanted to help, each on a single six-point item, and reported how much gratitude they would feel; the three ratings fitted well as indicators of a single latent benefit appraisal, which accounted for 83% of the variance in state gratitude once measurement error was removed.

The person who helps and the person who is helped give different answers about the same event, and this has been recorded on all four dimensions used in our current study, though never more than one or two at a time. Regarding the willingness of the helper, the evidence concerns anticipation rather than hindsight. To test whether the difference arises from perspective itself, Flynn and Lake (2008) randomly assigned participants to read the same scenarios as either the person asking or the person who might help, and found that those on the helper’s side estimated that 49.6% of people would agree, while those on the asker’s side estimated only 34.3%; Bohns (2016) reviewed 12 studies covering 14,757 real requests and found the average underestimate to be 48%. Whether the same gap appears once help has already been given is a separate question. Regarding how difficult the help is, Newark et al. (2017) compared estimates of how much effort someone puts in when helping you against how much effort someone puts in when working for themselves, and found the underestimate only in the first case. Thus, it is not a general tendency to undervalue other people’s effort, but something specific to being on the receiving end. Concerning cost and benefit, Zhang and Epley (2009) found across six experiments that helpers expect to be repaid in proportion to what the help cost them, while what recipients actually give back reflects what they got out of it rather than what it cost. Regarding how much gratitude is felt, Flynn and Adams (2009), studying engagement rings, birthday gifts, and hypothetical scenarios, found that givers expect more expensive gifts to be appreciated more, while recipients’ appreciation is unrelated to price, because givers treat a higher price as a sign of greater thoughtfulness. Converse and Fishbach (2012) asked both sides at once: as two strangers worked through a task together, the person being helped felt less indebted once the task was finished, while the helper expected the opposite. Together, these studies cover all four dimensions used in our current study, but none measures them together, so the shape of the asymmetry across the four remains unknown.

The gap is not a fixed quantity. Zhao and Epley (2022), across six experiments with 2,118 participants, confirmed that people asking for help underestimate how willing others are, but could not reproduce the explanation Flynn and Lake (2008) provided for it. In one live interaction, the effect ran the other way, with askers overestimating rather than underestimating how uncomfortable a helper would feel saying no. Zhang and Epley (2009) found that when both sides were asked outright what repayment should be based on, recipients stated it should track what the help cost the helper, while helpers said it should track what the recipient received, which is the reverse of how each side actually behaved. Li and Liu (2025), testing 240 Chinese undergraduates on scenarios involving strangers, found that helpers underestimated how warmly the other person felt when the help succeeded but overestimated it when the help failed. Therefore, the size of the gap, and sometimes its direction, changes with the question asked, with the situation, and with whether the help worked. Measuring one dimension in one situation can only provide part of the picture.

The previous section calls for a case in which the same event is judged from both sides, and gratitude fits this criterion. The principle is not in dispute: help deserves thanks. However, how much help it was, and how much thanks it warrants, depends on which side is answering. We pose four questions regarding a single act of help: how willing the helper was, how much the other person needed it, how difficult it was to provide, and how much gratitude it warranted. Difficulty and gratitude follow directly from Tesser et al. (1968) and Wood et al. (2008), where the cost to the benefactor is one of the appraisals that drives felt gratitude. Need is our own operationalisation: whereas those studies asked how valuable the benefit was, we ask how much the person needed it, which is what value amounts to when what is given is help rather than a gift. Willingness has no direct precedent as an appraisal dimension, and the review by Flynn (2006) suggests it may work in the opposite direction, since a helper who seems glad to help is credited with less. Two of the four questions therefore concern the person helping, and two concern the person helped.

### 1.3 Can we measure how differently the two sides interpret the same situation?

To find out how far helpers and those they help differ in interpreting the gratitude a single episode warrants, the situation itself can serve as a probe: change one component of it and observe how each side responds. Several such probes have been used, but rarely with both sides asked at once.

The first probe is how urgently the help was needed. Flynn (2006), reviewing what is known about how people evaluate help at work, argued that the same urgency means opposite things to the two sides: the more pressing the recipient’s need, the more grateful they will feel once it is met and the more willing they will be to reciprocate, whereas to the helper, a pressing need makes the help appropriate rather than extraordinary, so the helper feels less entitled to anything in return. Peng et al. (2018), wanting to know whether gratitude and indebtedness serve separate functions in social exchange, had participants read a scenario in which a colleague drives them home after they miss the last bus, considering the cost of the favour, with the colleague either living the same way or having to make a long detour, against its urgency, with the participant either about to miss a reservation for their partner’s birthday or in no particular hurry, and collected ratings of gratitude, indebtedness, the obligation to repay, and the wish to be near the colleague. The cost manipulation worked; the urgency manipulation did not, since participants rated the favour as highly beneficial even when nothing was pressing, and the authors report that they could therefore neither support nor reject any relation between urgency and gratitude. Peng et al. (2018) also found that cost predicted indebtedness while benefit and intention predicted gratitude, and that once both emotions were entered together, gratitude no longer predicted the obligation to repay on its own. Taken together, the two halves of the reversal Flynn (2006) described sit in two different systems, the recipient’s in gratitude and the helper’s sense of entitlement in indebtedness, so the probe yields no results unless the same question is posed to both sides.

A second probe is who initiated the help, and here the existing work is scattered. Lee et al. (2019), wanting to separate what reactive helping and proactive helping each bring to the helper, ran 2 weeks of daily surveys with full-time employees and found that helping in response to a request was associated with an increase in the gratitude helpers received, while helping without being asked was not; they then had separate samples of helpers and recipients each recall one episode of each kind and found that recipients reported expressing more gratitude for help they had asked for than for help offered unprompted, while helpers reported almost the same amount of gratitude received either way. Peng et al. (2018), in their autobiographical recall study, found the direction reversed, with offered favours eliciting more gratitude than requested ones, though only marginally. Shang et al. (2021), wanting to know whether helpers misjudge recipients when their help makes things worse, directly crossed the two forms in a parking scenario with Chinese participants and found that the form of the help made no difference anywhere in the design. Harari et al. (2022), wanting to separate anticipatory from reactive helping in the workplace, showed across four studies that help offered in advance of being asked is more self-threatening to recipients, thereby lowering their willingness to accept it as well as their evaluations of the helper’s performance and the relationship, an effect stronger when the helper outranked them; but what they measured was threat and evaluation, not gratitude. Four designs, three directions: more, less, and no difference, each obtained from one side and about a different aspect.

The study closest to the present design is Converse and Fishbach (2012), because it is among the few that had both sides answer the same question at the same moment. Wanting to know when over the course of receiving help beneficiaries feel most appreciative, they had unacquainted participants work in pairs on a data-entry task, one reading the data aloud and the other typing it, so that the reader was the helper and the typist the beneficiary, and collected ratings twice, once while the task was underway and once a few minutes after it was finished: beneficiaries reported how much they felt they owed their partner, and helpers estimated how much their partner felt they owed. Beneficiaries felt less indebted once the task was over, while helpers expected the opposite, and the two judgements moved reliably apart. One change to the situation, and the two sides went in opposite directions, which is visible only because both were asked.

Taken together, the situation is not merely a backdrop but a probe that can illuminate the differences between the two sides; for the probe to yield any insights, though, the same event has to be judged by both sides, at the same moment, on the same question. Existing work has used one probe at a time, has mostly asked one side, and has measured one or two dimensions. The four situational conditions used in our current study, therefore, apply both probes to the same event: need is varied between a pressing case and one that has already been met, giving the Pressing and Unneeded conditions, and how much the helper contributes is varied between help that meets the minimum the situation demands and help that goes beyond it, giving the Baseline and Considerate conditions.

### 1.4 Does telling a model who it is, or writing in another language, change how it answers?

Interpreting a scenario can be assigned to a model through an API rather than a chat window. One advantage is that every request is independent, with no memory of anything that came before. More importantly, each component of the prompt can be varied on its own, so it becomes possible to test what is driving the model’s answers. The definition of an identity is one such component. As noted in Section 1.1, Salewski et al. (2023) found that adding nothing more than “If you were a …” shifted model behaviour across several tasks. In the same spirit, Zheng et al. (2024), wanting to know whether an identity in the system prompt improves performance on objective tasks, assembled 162 roles spanning interpersonal relationships (mother, roommate, colleague, mentor) and professional domains (lawyer, nurse, software engineer, statistician), had four families of models answer 2,410 multiple-choice questions, and fitted mixed-effects regressions controlling for differences between models; no role performed reliably better than the control condition with no role at all, and in some models, most roles had a detrimental effect. They then tried to select the best role for each question automatically and found that every selection strategy performed only marginally better than picking a role at random, concluding that the effect of an identity may be largely unpredictable. Hu and Collier (2024) tested the same concept on subjective ratings, providing models with an annotator’s demographic and attitudinal information and asking them to predict that annotator’s ratings of offensiveness, politeness, and irony. Adding the information helped, but only slightly, and how much it helped depended on whether that information could predict human ratings on the task in the first place: on survey data where demographics predict how a person votes, the best model captured about eight-tenths of what was predictable, whereas on most annotation tasks, where demographics account for little of the disagreement between annotators, stating an identity did almost nothing.

How the identity is written matters as well. Lutz et al. (2025) identified two axes of variation from 47 simulation studies: the format in which the role is adopted (direct assignment, third person, interview) and the way the demographic cue is presented (a name, an explicit description, a structured list of attributes). They crossed these axes across five open-source models (Llama-3.3-70B, Llama-3.1-8B, OLMo-2-32B, OLMo-2-7B, Gemma-3-27B). The interview format and name-based cueing produced fewer stereotyped words, more semantic diversity, and better alignment with human opinion, while direct assignment paired with an explicit description was among the weaker combinations. The present study uses direct assignment, so its findings should be regarded as a lower bound.

The other component that can be varied is the language of the materials. Models do judge differently across languages, and those differences do not necessarily stem from cultural factors. Hämmerl et al. (2023), wanting to know whether multilingual models impose English moral norms on other languages, compared the moral judgements of a multilingual model against monolingual models in Arabic, Czech, German, English, and Chinese. They examined the sentence pairs in German-English and Czech-English parallel subtitles whose scores diverged most. Almost all of these divergences stemmed from a single word being misread: the German *Gift* (poison) was scored positively, presumably because it resembles the English *gift*, and the English “Traitors … like you!” was scored positively because “like you” was interpreted as a good thing. By applying the Moral Foundations Questionnaire to the same models, they found that the scores differed across languages but did not correspond to the cultural differences observed among human respondents from the matching countries. Models respond differently in different languages, then, and what they rely on in doing so is largely lexical.

### 1.5 The current study

The present study tests how far large language models reproduce the benefactor and beneficiary asymmetry humans show in judging gratitude, and what separates the models that come closest from those that do not. We gave 48 Chinese university students and 12 models an identical task: 24 everyday helping scenarios, each written in four situational versions and two role versions, on which both rated willingness, need, difficulty, and gratitude. Willingness and difficulty were always asked about the person providing help, while need and gratitude concerned the person receiving it, so reversing the role reverses whether a rating reports one’s own state or estimates someone else’s (Flynn and Adams, 2009; Zhang and Epley, 2009). Models were queried through their providers’ APIs, with each text being processed 30 independent times, and without conversational context. We used Bayesian mixed-effects models throughout to answer three questions.

- RQ1: When humans read the same helping scenario as the person receiving help or as the person giving it, how do the four situational versions affect their judgements of willingness, need, difficulty, and gratitude?
- RQ2: How far do language models show the same pattern, and does it depend on the model’s capability tier or on whether its provider is based in China or the West?
- RQ3: Does writing the scenarios in English, or stating an identity in the prompt, bring a model closer to the human pattern?

## 2 Materials and methods

### 2.1 Materials

The materials comprised 24 everyday helping scenarios written for this study and set on a Chinese university campus, such as lending an umbrella to a deskmate caught in a downpour or waking a roommate who had overslept. Each scenario was written in four situational versions and two role versions, resulting in 192 texts in total (24 × 4 × 2). All were written in Chinese and followed the same structure: a passage of narrative context concluding with a single utterance by the person providing the help. Table 1 presents one scenario in full.

**Table 1:** Sample stimulus set for one of the 24 scenarios.

Condition	Beneficiary version (Form A)	Benefactor version (Form B)
Baseline	After class it suddenly starts pouring with rain, and you do not have an umbrella. You ask your deskmate whether you could walk together, and he agrees: “*Let’s go together*.”	After class it suddenly starts pouring with rain, and your deskmate does not have an umbrella. He asks you whether you could walk together, and you agree: “*Let’s go together*.”
Considerate	After class it suddenly starts pouring with rain, and you do not have an umbrella. Your deskmate, who is just about to leave the classroom, notices this and stops to share his umbrella with you without being asked, saying: “*Let’s go together*.”	After class it suddenly starts pouring with rain, and your deskmate does not have an umbrella. As you are just about to leave the classroom you notice this, and you stop to share your umbrella with him without being asked, saying: “*Let’s go together*.”
Pressing	After class it suddenly starts pouring with rain, and you do not have an umbrella. You are in a hurry to get back to your dormitory to fetch something, so you ask your deskmate whether you could walk together, and he agrees: “*Let’s go together*.”	After class it suddenly starts pouring with rain, and your deskmate does not have an umbrella. He is in a hurry to get back to the dormitory to fetch something, so he asks you whether you could walk together, and you agree: “*Let’s go together*.”
Unneeded	After class it suddenly starts pouring with rain and you do not have an umbrella, but you are not planning to leave the classroom any time soon. Your deskmate, unaware of this, offers to share his umbrella with you, saying: “*Let’s go together*.”	After class it suddenly starts pouring with rain and your deskmate does not have an umbrella, but he is not planning to leave the classroom any time soon. Unaware of this, you offer to share your umbrella with him, saying: “*Let’s go together*.”
Rating questions, identical across the four conditions	How willing do you think your deskmate is to provide this help? How much do you think you, the recipient, need this help? How difficult do you think it is for your deskmate to provide this help? How grateful do you think you, the recipient, feel toward your deskmate?	How willing do you think you are to provide this help? How much do you think your deskmate, the recipient, needs this help? How difficult do you think it is for you to provide this help? How grateful do you think your deskmate, the recipient, feels toward you?

The scenario texts are the English versions used in the language manipulation of Section 3.5; the human participants read the Chinese originals. The rating questions are presented here in a closer rendering of the Chinese, which explicitly marks the recipient. Baseline and Considerate differ in how much the helper invested. Pressing retains the request but makes the need urgent; Unneeded removes the need, and with it the occasion to ask, so the help is offered.

The four situational conditions consist of a baseline and three variations. The baseline features the recipient asking for help and the helper agreeing. The Considerate condition has the helper doing more than the situation minimally requests. The Pressing condition makes the recipient’s need urgent. The Unneeded condition presents help that provides no value because the need had already been met, did not exist in the first place, or had lapsed by the time the help arrived. The two role versions placed the reader either as the person giving help or as the person receiving it, and after each text, participants answered the same four questions, shown at the foot of Table 1. The situation was manipulated within participants and the role was varied between them: each participant read all 24 scenarios in all four situational conditions, totalling 96 texts, but in only one of the two role versions.

The Considerate condition contrasts help that exceeds the minimum the situation requires with help that meets it. This is realised in two ways: in 11 scenarios, the helper acts before being asked, and in 13, the helper does more than was requested. We do not treat these as a single construct, so their difference is captured by the by-scenario intercept and slope rather than absorbed into the fixed effect, and the considerate effect reported below is an average over both. This interpretation is exploratory: we arrived at it after examining the materials, and the pattern of results is consistent with it rather than a test of it. The contrast influenced willingness (0.402) and difficulty (0.422), the two questions about the helper, while leaving need untouched, which is where a manipulation of the helper’s investment would be expected to have an effect. The contrast between Pressing and Unneeded does not isolate a single component either, since a person who does not need help has no reason to ask for it, so we interpret it as the joint effect of an absent need and unrequested help. The two contrasts are reported separately throughout.

In some scenarios, switching perspective also meant switching counterpart. In 16 of the 24 scenarios, the counterpart was a peer, such as a deskmate, a roommate, a friend, or a stranger, so that reversing the role left that person’s identity and standing unchanged. In the remaining 8, the relationship could not be reversed, since a student does not supervise their own advisor, and the counterpart changed along with the role, with an academic advisor becoming a junior student and a class advisor becoming a roommate. Whether the role effect is driven by this change of counterpart is tested in Section 3.7.

### 2.2 Human participants

Forty-eight university students from universities in the Songjiang district of Shanghai provided analysable data (59 took part; 11 were excluded: six for implausibly short completion times, one for a long idle period, and four whose questionnaire submissions could not be matched to their rating data). Participants were randomly assigned to one of the two role versions. Group A (*n* = 24; 11 male, 13 female; *M*age = 22.96, *SD* = 2.96) read the beneficiary version and Group B (*n* = 24; 12 male, 12 female; *M*age = 22.04, *SD* = 2.80) read the benefactor version; the two groups did not differ in gender composition (Fisher’s exact test, *p* = 1.000) or age (Welch’s *t*(45.9) = −1.10, *p* = 0.276).

Participants completed the study online through https://www.wenjuan.com/. Within each form, the 96 texts were presented in a fixed pseudorandom order, constructed so that the four conditions of one scenario never appeared consecutively; the two forms were randomised independently, so a given serial position corresponds to different scenarios in Form A and Form B. The study was approved by the ethics committee of the Institute of Language Sciences, Shanghai International Studies University, and all participants gave informed consent.

Participants also completed four questionnaires after the rating task: the 20-item Toronto Alexithymia Scale (Bagby et al., 1994), the Interpersonal Reactivity Index (Davis, 1980), the Liebowitz Social Anxiety Scale (Heimberg et al., 1999), and the 60-item BFI-2 (Soto and John, 2017; Zhang et al., 2022). These measures do not enter into any model reported below. They are described here and compared across the two groups in Supplementary Table S11. Internal consistency computed on the present sample ranged from *α* = 0.60 to 0.93; four subscales fell below 0.70 (TAS-EOT *α* = 0.60, IRI-PT *α* = 0.61, TAS-DDF *α* = 0.63, IRI-PD *α* = 0.65), and the low value for IRI perspective taking is one reason, along with the sample size, that we do not use these measures as predictors of the role effect.

The two groups did not differ on any of the 14 non-redundant trait indices (all *p* ≥ 0.123, all Holm-corrected *p* = 1.000). On the two dispositions most directly relevant, the groups were similar: agreeableness, *d* = −0.24, *p* = 0.414; empathic concern, *d* = −0.01, *p* = 0.976. The largest imbalance was in neuroticism, *d* = −0.45, 95% CI [−1.03, 0.12], with Group B scoring higher. These comparisons are descriptive. With 24 per group, the smallest detectable standardised difference at conventional power is *d* = 0.83, so they can rule out a gross imbalance but nothing finer; group equivalence here rests on random assignment rather than on these tests.

### 2.3 Language models and querying procedure

Querying through an API keeps every request independent and stateless: the model carries no conversational history and has never seen the other conditions of the same scenario or the other role version. We queried six providers, three based in China (DeepSeek, Alibaba, ByteDance) and three based in English-speaking countries (Anthropic, OpenAI, Google), taking the flagship and the light model from each, 12 in total (Table 2). Every model received all 192 texts, each queried 30 times, for 5,760 calls per model and 69,120 in the main experiment. The prompt reproduced word for word what the human participants had read: the same narrative context, the same four questions, the same 5-point scale.

**Table 2:** The 12 language models.

Provider	Model	Tier	Origin	API identifier
Anthropic	Claude-Opus-4.5	flagship	Western	claude-opus-4-5-20251101
Anthropic	Claude-Haiku-4.5	Light	Western	claude-haiku-4-5-20251001
OpenAI	GPT-5.4	flagship	Western	gpt-5.4-2026-03-05
OpenAI	GPT-5.4-mini	Light	Western	gpt-5.4-mini-2026-03-17
Google	Gemini-3.5-Flash	flagship	Western	gemini-3.5-flash
Google	Gemini-3.5-Flash-Lite	Light	Western	gemini-3.5-flash-lite
DeepSeek	DeepSeek-Pro	flagship	Chinese	deepseek-v4-pro
DeepSeek	DeepSeek-Flash	Light	Chinese	deepseek-v4-flash
Alibaba	Qwen3.7-Max	flagship	Chinese	qwen3.7-max-2026-06-08
Alibaba	Qwen3.7-Flash	Light	Chinese	qwen3.7-flash-2026-07-15
ByteDance	Doubao-2.1-Pro	flagship	Chinese	doubao-seed-2-1-pro-260628
ByteDance	Doubao-2.1-Turbo	Light	Chinese	doubao-seed-2-1-turbo-260628

Tier distinguishes each provider’s more capable model from its lighter, faster counterpart at the time of querying and is relative within a provider rather than absolute across them. Origin records where the provider is based; no provider publishes the composition of its corpus, so the column is not a measurement of it. Identifiers are the exact strings sent to each provider’s API. All models were queried between 3 and 5 August 2026; per-model query windows are deposited with the data.

All calls used temperature 1.0 and top_p 1.0, with no system prompt outside the persona condition and no conversational context; every request was independent and stateless, and the logged parameters recorded 1.0 for both across all three batches. Extended reasoning was disabled throughout. Four providers required an explicit setting: thinking.type = “disabled” for DeepSeek and ByteDance, enable_thinking = false for Alibaba, and thinking_budget = 0 for Google. OpenAI and Anthropic models had it off by default and needed no parameter. We disabled it because each provider documents that temperature and top_p are ignored while reasoning is active, which would have undercut the sampling design behind the 30 independent runs per text. Where a provider reports a reasoning token count, it was zero for every call; Alibaba, Anthropic, and Google do not expose the field, so for those six models, the setting is the only record.

The whole procedure was then repeated with a single sentence supplied as a system message, 你是一名中国大学生。请以你自己的真实感受作答 (“You are a Chinese university student. Answer according to your own genuine impressions”), adding a further 69,120 calls; nothing else changed, and the zero-shot condition used no system message at all. This tests whether the role effect under zero-shot prompting is weak simply because the prompt never asked the model to adopt a perspective. The sentence supplies both an identity and an instruction to answer in the first person, and it arrives through the channel each provider reserves for role setting, so this is a strong rather than a minimal version of that manipulation.

We also varied the language of the materials. The 192 Chinese texts were translated into English by Claude Opus 5 (high); GPT-5.6 Sol (thinking effort: high) reviewed the two versions side by side and proposed revisions, which Claude reconsidered and incorporated, and the result was checked by the first author, a native speaker of Chinese with English at CEFR C1. This English set was sent to the six models of the three Chinese providers, adding 34,560 calls.

### 2.4 Statistical analysis

All analyses are Bayesian mixed models fitted with brms (Bürkner, 2017), and all model formulas are listed in Supplementary Table S1. We chose this framework because several of the questions here concern whether an effect is absent or too small to matter, rather than whether it differs from zero. A posterior interval answers the former question directly, while a significance test does not. Additionally, the comparison relies on placing model estimates against a human benchmark that is itself uncertain, which the posterior of the human effect naturally accommodates. The same approach was used by Chen et al. (2026).

*Data, coding, and the unit of analysis.* Five sets of analyses follow, and they do not all use the same data. (1) The human benchmark employs the human ratings alone. (2) The comparison of each model against the humans utilises the human ratings together with one model’s Chinese zero-shot runs, fitted 12 times, once per model. (3) The language analysis uses model data only, comparing each model’s Chinese and English zero-shot runs across the six models that have both. (4) The persona analysis also uses model data only, comparing each model’s zero-shot and persona runs on the Chinese materials across all 12. (5) The moderator analysis uses no ratings at all but incorporates the 12 role effects estimated in (2).

We coded situation, role, and agent to sum to zero. Under treatment coding, the role coefficient would report the role effect in the reference situation alone rather than the average over all four. Therefore, we employed emmeans contrasts (Lenth et al., 2018), which return differences in raw scale points, instead of reading them off the coefficient table.

We ran the models 30 times on each text, and we treat those 30 runs as 30 draws from one model under one prompt, rather than as 30 simulated participants: they resemble each other far more closely than 30 individuals would. The corresponding unit on the human side is a person, and there were 48. What we ask of the model side is, therefore, whether a model’s average rating shifts when the role label changes.

We also keep each text’s serial position in the questionnaire as a random effect, since participants worked through 96 texts in one sitting, and later answers may differ from earlier ones. We randomised the two forms separately, so position 40 represents a different scenario in Form A than in Form B. On the model side, the term means nothing, since each call is independent and unordered.

*The human benchmark.* We fitted a Bayesian mixed model to the human ratings for each of the four questions, with situation, role, and their interaction as fixed effects. Model selection used leave-one-out cross-validation across four candidates: participant alone; participant and scenario; those plus serial position, entered as item; and those plus a by-scenario random slope for role. The winning structure retained the by-scenario slope (ΔLOO = 27.0 against the next best; Supplementary Table S2), which means the role effect varies across scenarios: rating ~ condition_c * role_c + (1 | pid) + (1 + role_c | scenario_id) + (1 | item). Two things about this selection should be noted. We ran it on the gratitude question alone and applied the result to all four, and we did not propagate the selection uncertainty into the intervals reported later, which are conditional on the structure chosen.

This stage provides the role effect and the two situational contrasts on each question, reported in Section 3.1.

*Each model against the humans.* We fitted each model jointly with the human data, using the structure selected above with agent and all its interactions added: rating ~ condition_c * role_c * agent_c + (1 | pid) + (1 + role_c | scenario_id) + (1 | item). Before applying that structure to all 12, we re-checked it on one model, GPT-5.4, since 30 runs of one model have a different shape from 48 participants; the same comparison selected the same structure there.

Two quantities are of interest. Situation × agent indicates how far a model’s sensitivity to the situational manipulation departs from that of the humans, and role × agent indicates how far its sensitivity to role departs. We also extracted each model’s own role effect separately, since a model can differ credibly from the humans and still show a substantial asymmetry.

Role is between participants on the human side and within runs on the model side, so a by-participant random slope for role is estimable for the models and not for the humans. We gave neither side one, since fitting it where it can be fitted and not where it cannot would confound role × agent with a difference in structure.

We used the same fits for two further breakdowns: the role effect computed within each of the four situations and the whole comparison repeated on each of the four rating questions. These are reported in Sections 3.2 and 3.4.

*What predicts a model*’*s role effect.* Capability tier and provider origin are properties of a model rather than of a rating, so fitting them to the ratings themselves would mean a four-way interaction across the whole data set and would add no information about the moderators. We instead entered the 12 role effects from the previous stage into a second-stage meta-regression, weighting each by its standard error so that a precisely measured model counts for more than a roughly measured one: *estimate | se(se) ~ tier + origin + (1 | obs)*. Provider was not entered: 12 observations over six providers give two points each, and each provider contributes exactly one flagship and one light model, so tier absorbs most of that dependency. Slope priors were Normal(0, 0.3), tighter than those used on the ratings, because 12 points cannot overrule a diffuse prior. Residual heterogeneity enters through a per-observation random effect, with the random-effects meta-analysis variance expressed in this framework.

We report no information criteria for these models. Every observation carries its own random effect, so removing one leaves that effect identified by the prior alone, and importance sampling breaks down, with Pareto *k* values reaching 1.07. The quantity that answers the question is the between-model standard deviation τ, the spread of true role effects once each estimate’s own measurement error is removed; a moderator that matters shrinks it. Results are reported in Section 3.3.

*The two prompt manipulations.* English marks person on every clause where Chinese can leave it to word order and context, and the zero-shot prompt never states an identity. Both manipulations were fitted to model data alone: rating ~ condition_c * role_c * language + (1 | pid) + (1 | scenario_id); rating ~ condition_c * role_c * persona + (1 | pid) + (1 | scenario_id). These omit the by-scenario slope for role that the joint models carry, so their intervals are narrower than the design supports, and we read the two results from the direction and the range of the estimates rather than from counts of credible intervals. Reported in Sections 3.5 and 3.6.

*Robustness, sampling, and software.* Four checks ask whether the results depend on choices made in the design or the analysis. First, scenario type (peer against status gap) and its interaction with role were added and fitted separately for the human data and for each model, keeping both scenario terms in the random effects so that the contrast is tested against variation between the 24 scenarios rather than against residual variation; the symmetry main effect is consequently absorbed by the scenario intercept, and only the interaction is interpretable. Second, three models spanning the observed range were refitted with a cumulative probit likelihood. Third, the joint model for GPT-5.4 was refitted under a tighter and a wider slope prior. Fourth, the equivalence classification was recomputed with the bound built from each end of the human 95% interval. (Reported in Section 3.7.)

Effects are reported in raw scale points rather than standardised: the humans and the models used the same five-point scale, and dividing each by its own response standard deviation would inflate the models’ effects and close part of the gap that the comparison addresses. We provide the posterior mean, the 95% highest density interval, and the probability of direction, and label an effect as credible when its interval excludes zero. Role effects are additionally interpreted against a region of practical equivalence set at half the human effect on the same question, computed from the raw group means rather than from the fitted posterior: the human design is balanced, so the raw difference estimates the same quantity without inheriting the uncertainty of the structure selection mentioned above. For gratitude, this bound is ±0.379. It assesses not whether an effect is zero but whether it is too small to count as the asymmetry shown by the humans, and it derives from the human benchmark without depending on any model result. Against it, we classify a role effect as above the region when its entire interval lies beyond the upper bound, equivalent when its entire interval lies inside, and not placed when the interval crosses a bound.

Priors were Normal (0, 1) on slopes, Normal (3, 1) on the intercept, and Exponential (1) on variance components, based on the reasoning that an effect of one whole point on a five-point scale is already very large. Sampling used 8 chains of 3,000 iterations with 1,500 warmup, giving 12,000 posterior draws, with adapt_delta = 0.95 and max_treedepth = 12; the meta-regressions used adapt_delta = 0.999 and max_treedepth = 15, since 12 observations with one random effect each produce a funnel geometry at the default settings. Every model is cached against a fingerprint of its formula, family, priors, sampler settings, contrast matrices, and data, so that a change to any of these triggers a refit. Analyses were carried out in R 4.3.3 (R-Core-Team, 2024) using brms 2.22.0 (Bürkner, 2017), emmeans 1.11.0 (Lenth et al., 2018), bayestestR 0.17.0 (Makowski et al., 2019), loo 2.8.0 (Vehtari et al., 2017), and tidybayes 3.0.7 (Kay, 2023).

## 3 Results

### 3.1 The human benchmark: beneficiaries and benefactors valued the help differently

Using Bayesian mixed models, we compared the ratings of two groups of 24 participants who read the same 24 scenarios, one positioned as the benefactor and the other as the beneficiary (Figure 1; cell means in Supplementary Table S4, contrasts in Supplementary Table S3). Two situational contrasts are reported for each question: Considerate against Baseline, which adds a helper who does more than the situation minimally requires without being asked, and Pressing against Unneeded, which sets a pressing need against one already met. An effect is deemed credible when its 95% highest density interval excludes zero; effects whose intervals include it are reported with *pd* alone.

**Figure 1:**
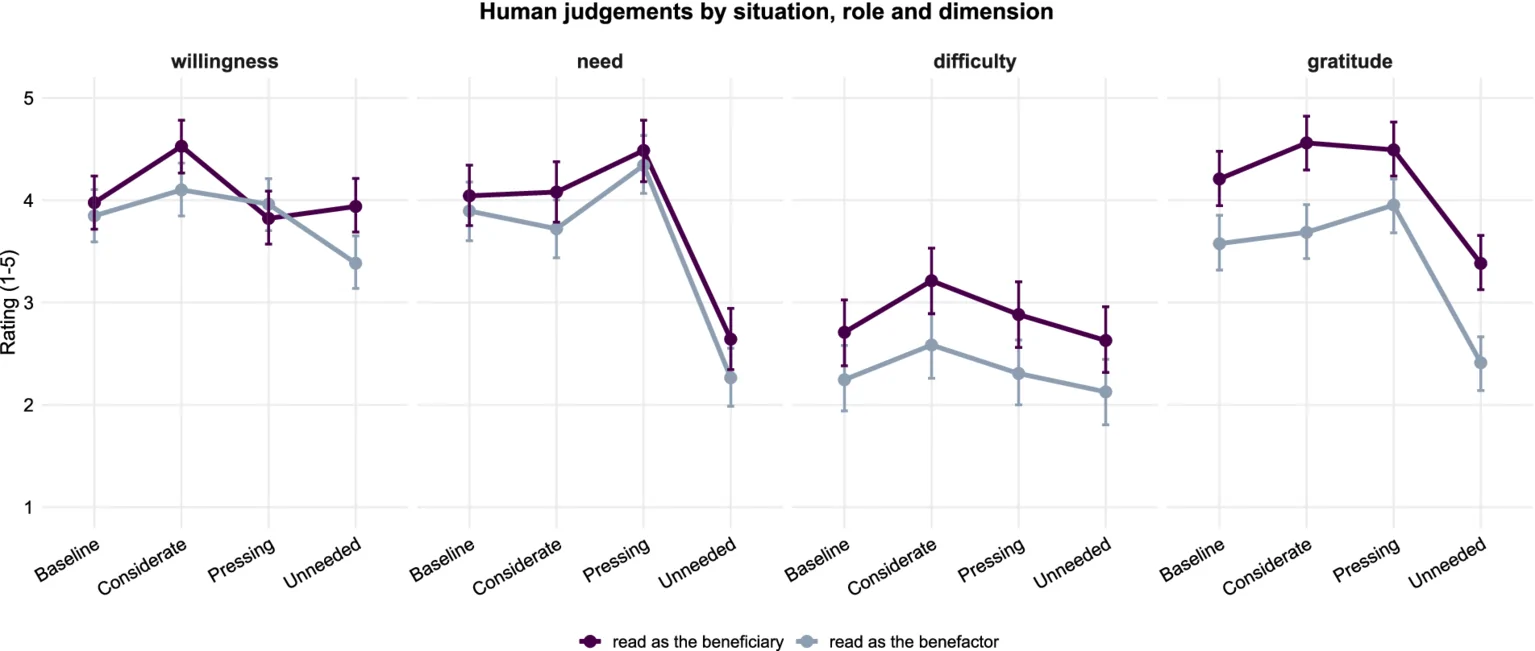
Judgements by 48 humans who each read 24 everyday helping scenarios in four situational versions, from one of the two sides. Marginal means with 95% highest density intervals, averaged over scenarios. Role was between participants: each person read only one version, so the two lines in each panel come from different groups of 24. Cell means are given in Supplementary Table S4 and the contrasts in Supplementary Table S3.

For willingness, how willing the helper was to provide assistance, the two sides did not differ significantly (0.243, 95% HDI [−0.054, 0.538], *pd* = 0.946). Ratings rose in Considerate compared to Baseline (0.402, 95% HDI [0.295, 0.527]) and rose less in Pressing compared to Unneeded (0.228, 95% HDI [0.118, 0.333]).

For need, how much the recipient needed assistance, the two sides again did not differ significantly (0.259, 95% HDI [−0.013, 0.546], *pd* = 0.964). Ratings rose steeply in Pressing compared to Unneeded (1.961, 95% HDI [1.862, 2.067]) and did not change in Considerate compared to Baseline (*pd* = 0.890). This is a treatment effect on one of the four rating dimensions, not an independent manipulation check, which we did not collect.

For difficulty, how hard it was for the helper to provide assistance, the two sides diverged: the side estimating another’s effort attributed more of it than the side reporting its own (2.86 against 2.32, 0.540, 95% HDI [0.160, 0.908], *pd* = 0.998). Ratings again rose more in Considerate compared to Baseline (0.422, 95% HDI [0.331, 0.518]) than in Pressing compared to Unneeded (0.216, 95% HDI [0.124, 0.306]).

For gratitude, how grateful the recipient felt towards the helper, the two sides diverged the most, by the largest margin of the four questions: the side reporting its own feelings rated them higher than the side estimating that feeling in another (4.16 against 3.41, 0.756, 95% HDI [0.422, 1.087], *pd* > 0.999). Ratings again rose steeply in Pressing compared to Unneeded (1.325, 95% HDI [1.241, 1.408]) and less in Considerate compared to Baseline (0.232, 95% HDI [0.147, 0.322]).

Three points can be drawn from these results. First, role changed how much the help was valued, but not how the two people in it were perceived: it affected difficulty and gratitude, but did not significantly affect willingness or need. Second, each situational contrast influenced the judgements about the party it describes the most. What the helper contributed describes the helper and most influenced willingness and difficulty; need describes the recipient and most influenced need and gratitude. The cross-over effects were smaller but mostly significant, so the two contrasts are not selective, only differently weighted; the one exception is that the helper contrast left need unaffected. Third, situation influenced these ratings more than role did on three of the four questions, the exception being difficulty, where the role effect (0.540) exceeded both situational contrasts. On gratitude, the need contrast was 1.325 compared to a role effect of 0.756. Both are what the models are asked to reproduce below, and the human role effect is also what they are measured against: the region of practical equivalence used throughout is half that effect, computed from the raw group means, so ±0.379 on gratitude.

### 3.2 LLMs overshoot people on how pressing the need was and undershoot them on whose side it is judged from

Each of the 12 models was fitted jointly with the human data, so that one posterior yields two quantities: how far the model’s sensitivity to the situation departs from that of the humans, and how far its separation of the two roles departs (Figure 2; values in Supplementary Tables S5–S7). We report both, because a weak role effect on its own could simply mean the model never processed the scenario properly. The situational term shows whether it did.

**Figure 2:**
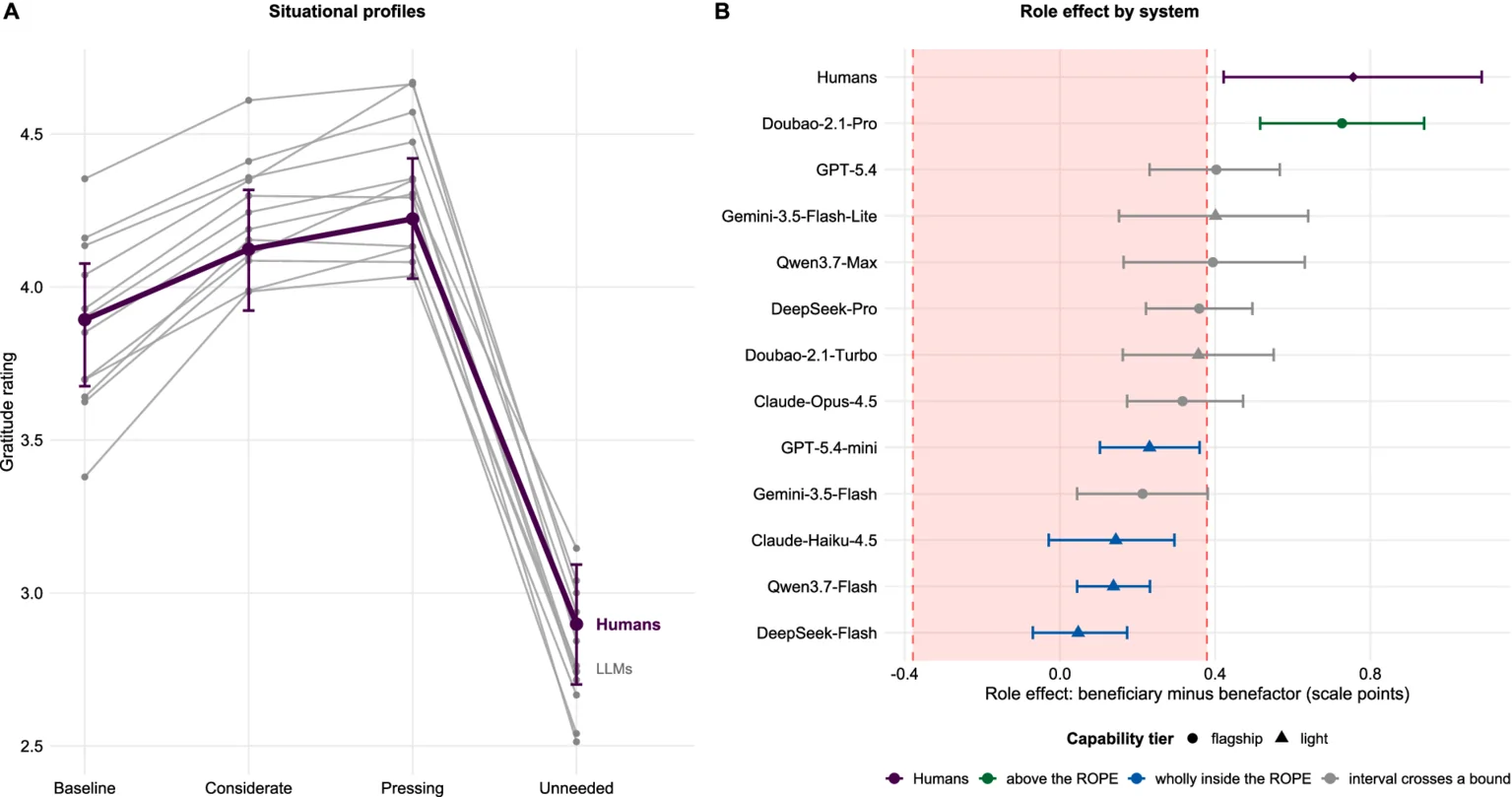
Gratitude ratings from 48 humans and 12 language models answering the same scenarios. **(A)** Ratings across the four situational conditions, one line per system. **(B)** The role effect with 95% highest density intervals; the shaded band is the region of practical equivalence, half the human effect. Shape marks capability tier; the human row is a diamond. Values in Supplementary Tables S5–S7.

For the situation, the models matched the humans and usually exceeded them (Figure 2A). The Pressing against Unneeded contrast was 1.325 in the human data and ranged from 1.159 (Doubao-2.1-Turbo) to 1.825 (Gemini-3.5-Flash) across the models. As a departure from the human contrast, it ranged from −0.071 (Doubao-2.1-Turbo) to 0.576 (Gemini-3.5-Flash), with 10 of 12 intervals lying entirely above zero. In Figure 2A, the model lines rise and fall with the human line across the four conditions, and most of them fall further between Pressing and Unneeded than the human line does.

For the role, every model gave a smaller beneficiary-minus-benefactor difference than the humans did, and how much smaller varied widely from one model to the next: from 0.047 (DeepSeek-Flash) to 0.727 (Doubao-2.1-Pro), compared to 0.756 in the humans (Figure 2B; Supplementary Table S5). Four of the 12 intervals lie wholly inside the region of practical equivalence, an asymmetry credibly smaller than half the human one, and one lies wholly above it; that count depends on where the bound is drawn, and Section 3.7 gives its range. Read as a difference from the human effect rather than as a value in its own right, the same quantity ranged from −0.685 (DeepSeek-Flash) to −0.022 (Doubao-2.1-Pro), and 11 of the 12 intervals fall entirely below zero (Supplementary Table S6). The exception is Doubao-2.1-Pro, whose difference included zero (−0.022, 95% HDI [−0.267, 0.224], *pd* = 0.57) and whose interval in Figure 2B overlaps the human interval along most of its length: on this contrast, that model is not distinguishable from the humans who took the same task.

Figure 3 plots the two departures against each other, one point per model, with the role difference from Supplementary Table S6 on the horizontal axis and the situational difference from Supplementary Table S7 on the vertical. They run in opposite directions and do not travel together. Gemini-3.5-Flash departs furthest on situation (0.576) and is also among the furthest on role (−0.519), whereas GPT-5.4 departs almost as far on situation (0.520) while being among the smallest role departures (−0.337); Doubao-2.1-Turbo is the only model below the humans on situation (−0.071) and is mid-range on role (−0.376). The Pearson correlation between the two columns is *r* = 0.08, though with 12 points, that estimate is too imprecise to rely on.

**Figure 3:**
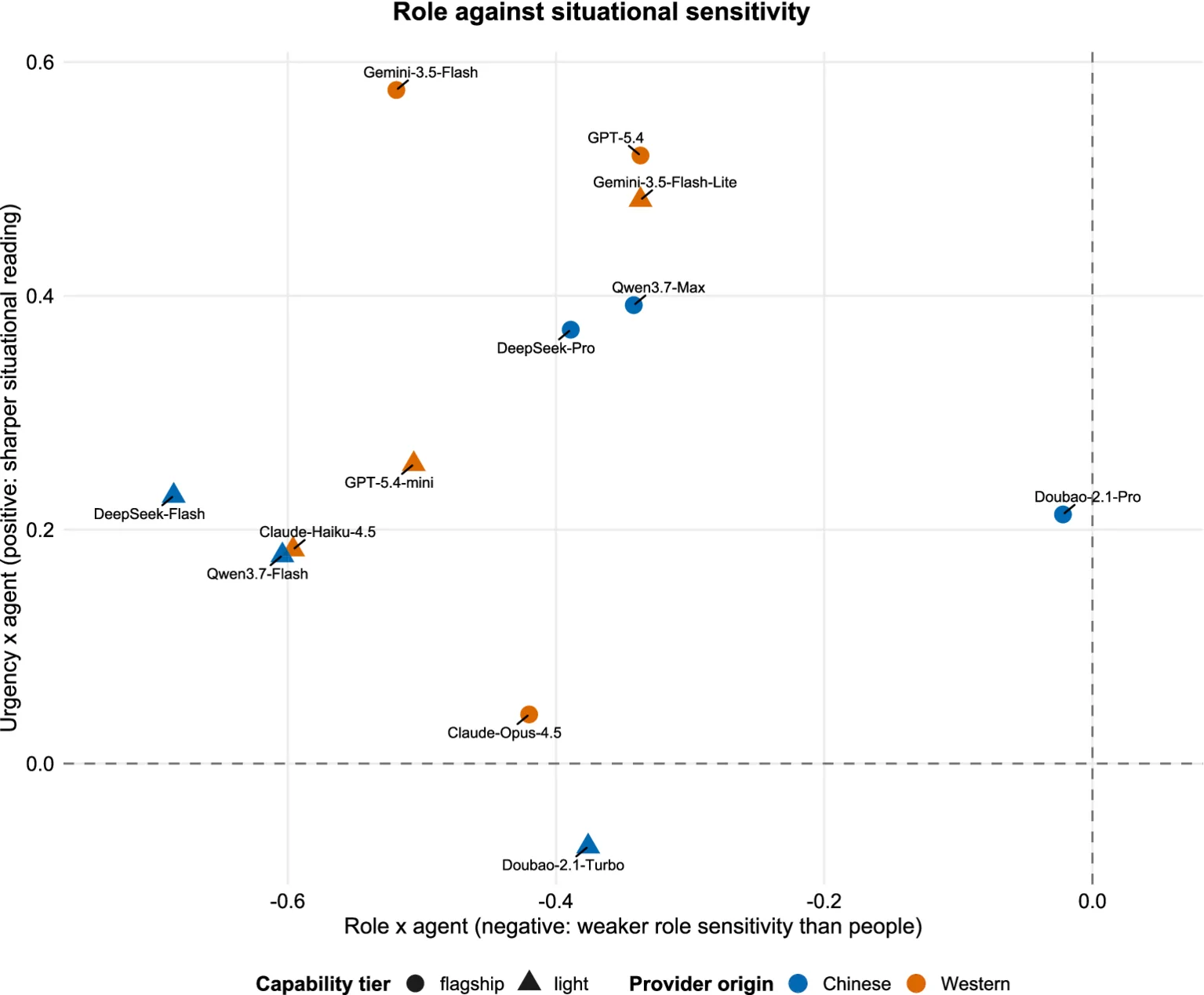
Each model’s departure from the human pattern on the two contrasts of Figure 2, one point per model: The role difference on the horizontal axis (Supplementary Table S6) and the situational difference on the vertical (Supplementary Table S7). Negative values on the horizontal axis indicate a weaker role effect than the human participants show; positive values on the vertical indicate a sharper situational contrast. Shape denotes capability tier, while colour indicates the provider’s country of origin, a proxy for the language that predominated during training.

### 3.3 Capability tier ordered the role effect; the provider’s country did not

The 12 role effects run continuously from 0.047 (DeepSeek-Flash) to 0.727 (Doubao-2.1-Pro) against a human value of 0.756, with no gap dividing them into two groups (Supplementary Table S5). The six flagship models averaged 0.402 compared to 0.220 for the six lighter ones, and adding tier to the meta-regression lowered between-model heterogeneity from τ = 0.159 (95% HDI [0.078, 0.265]) to τ = 0.120 (95% HDI [0.027, 0.236]). The contrast itself was 0.186, with an interval including zero (95% HDI [−0.023, 0.367], *pd* = 0.967; Supplementary Table S8). The same ordering is visible in Figure 2B: all four models whose intervals lie wholly inside the equivalence band are light-tier, and the one lying above it is a flagship. Three indicators therefore point in the same direction. The group means, the drop in τ, and the equivalence classification all order the two tiers similarly, while the contrast that would establish the difference does not exclude zero. With 12 observations in two groups of six, the meta-regression is not in a position to establish it; what the ordering supports is that tier is worth carrying into a larger sample, not that the difference is demonstrated here.

The provider’s country of origin does not determine the effect. Its contrast was −0.040 (95% HDI [−0.241, 0.148], *pd* = 0.670), and adding it raised heterogeneity to τ = 0.169 (95% HDI [0.081, 0.286]), so it accounts for none of the spread that tier partly accounts for (Supplementary Table S8). Both ends of the range are models from providers based in China: Doubao-2.1-Pro at 0.727 and DeepSeek-Flash at 0.047 (Supplementary Table S5). In Figure 3, the two colours are mixed along both axes. Had the shortfall come from training mainly on Western text, models from Chinese providers should have performed better as a group; they did not. Origin only records where a company is based, since no provider publishes what it trained on, and a null on that proxy is not a null on corpus composition. Section 3.5 poses the same question regarding the language of the materials rather than the provider.

### 3.4 Willingness is where people and models point in opposite directions; elsewhere they differ in size

For willingness, humans and models leaned in opposite directions (Figure 4A). The humans rated the helper as more willing when reading as the person being helped, though that estimate did not exclude zero (0.243, 95% HDI [−0.054, 0.538]). Nine of the 12 models leaned the other way, rating the helper as more willing when answering as the helper, with the range running from −0.177 (Gemini-3.5-Flash) to 0.120 (Gemini-3.5-Flash-Lite; Supplementary Table S5). Taken as a difference from the human effect, this runs from −0.409 to −0.116, with nine of 12 intervals entirely below zero (Supplementary Table S6). What is established here is therefore the departure between the two sides, not a reversal of two credible effects: the human estimate on this question is positive but not credible, so the claim is that the models sit credibly below it, not that people and models each show a reliable effect in opposite directions.

**Figure 4:**
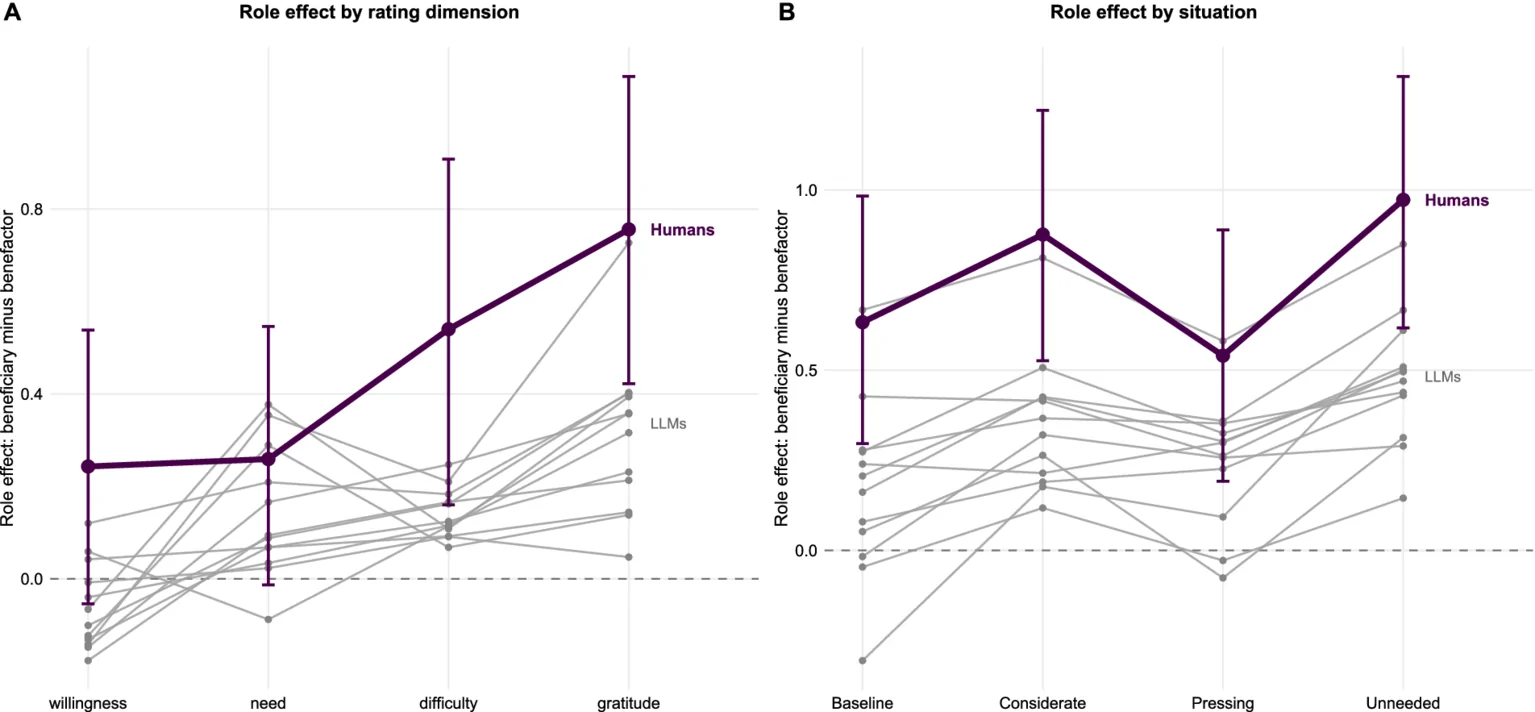
The role effect broken down in two ways, on the same beneficiary-minus-benefactor scale as Figure 2B. **(A)** The effect on each of the four rating questions, averaged over the four situational conditions; most models fall below zero on willingness, where the human estimate is positive but does not exclude zero. **(B)** The effect on gratitude computed separately within each of the four situational conditions. Purple represents the human data with its 95% highest density interval; grey lines are the 12 models, shown without intervals. Values for panel A are in Supplementary Tables S5, S6; those for panel B are given in the text above.

For need, both sides leaned the same way, rating it higher from the beneficiary’s position, and most models did not differ credibly from humans. Model effects ran from −0.088 (Claude-Opus-4.5) to 0.377 (Qwen3.7-Max) against a human 0.259 (Supplementary Table S5), with only three of 12 differences excluding zero (Supplementary Table S6).

For difficulty, every model also rated it higher from the beneficiary’s position, but all 12 rated the difference smaller than the humans did, from 0.068 (Qwen3.7-Flash) to 0.247 (Doubao-2.1-Turbo) against a human 0.540 (Supplementary Table S5), with 12 of 12 differences entirely below zero (Supplementary Table S6).

For gratitude, the same holds, with 11 of 12 below the human effect (Section 3.2). Willingness is therefore the only question on which the models turn in the opposite direction as a group; on need, one model does so alone, and on difficulty and gratitude, none does.

Within each situation separately (Figure 4B, gratitude only), the human role effect was largest under Unneeded (0.973, 95% HDI [0.618, 1.315]) and smallest under Pressing (0.540, 95% HDI [0.192, 0.889]): the two sides disagreed most about an episode in which the help was not really needed, and least about one in which it plainly was. The models traced the same shape at a lower level, peaking under Unneeded from 0.145 (DeepSeek-Flash) to 0.850 (Doubao-2.1-Pro) and bottoming under Pressing from −0.076 (Qwen3.7-Flash) to 0.581 (Doubao-2.1-Pro). The human line lay above every model line in all four situations, so the gap reported in Section 3.2 is not attributable to any one of them.

### 3.5 In English, the role effect rose for five of the six models tested in both languages

The 192 texts were translated and presented again to the six models of the three providers based in China, with everything else held constant, to examine what happens when the role is marked in the grammar: English marks person in every clause where Chinese can leave it to word order and context. The English-minus-Chinese difference ranged from −0.155 (Qwen3.7-Flash) to 0.224 (Doubao-2.1-Turbo), with five of the six models showing a larger role effect in English and five of the six intervals entirely above zero; the sixth, Qwen3.7-Flash, moved credibly in the opposite direction (Figure 5A; Supplementary Table S9). These fits omit the by-scenario random slope for role that the main comparison carries, so the intervals are narrower than the design supports, and the counts are provided for orientation; the direction and the range are what the argument rests on. The situational contrasts did not change with language, so this is specific to role rather than a general gain from translating into a better-resourced language.

**Figure 5:**
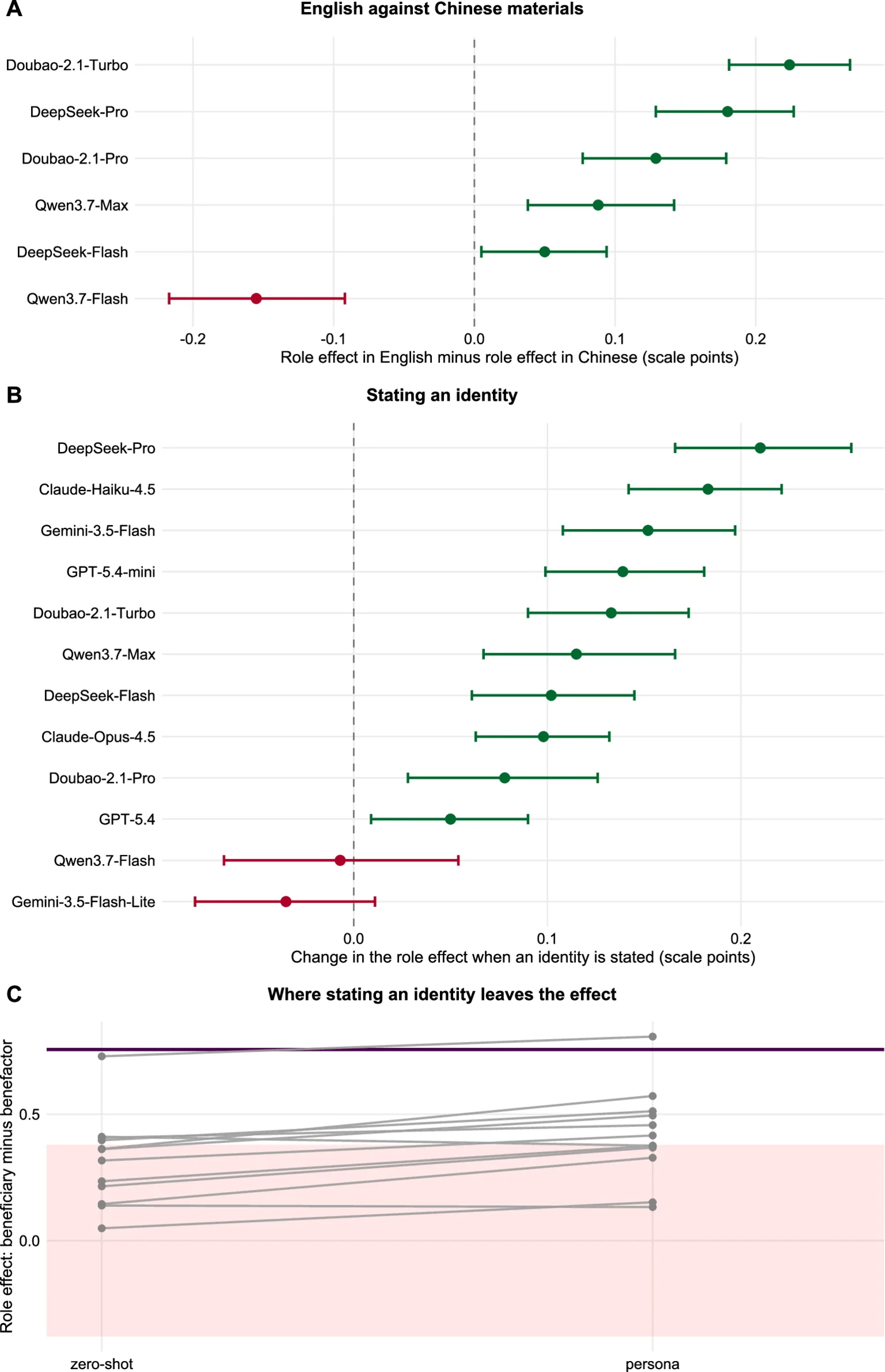
Two manipulations of the model side, both on gratitude. **(A)** The role effect with the scenarios in English minus the same effect with the scenarios in Chinese, for the six models of the three providers based in China (Section 3.5). **(B)** The change in the role effect when a system prompt names the model as a Chinese university student, 12 models (Section 3.6). **(C)** The same two prompting conditions as levels rather than as a difference; the horizontal purple line at the top is the human estimate of 0.756 and the shaded band is the region of practical equivalence. Points in A and B are posterior means with 95% highest density intervals, and colour marks the sign of the point estimate, so a model whose interval includes zero may still be coloured. Both were fitted without the by-scenario random slope for role carried by the main models, so their intervals are narrower than the design supports. Values in Supplementary Table S9.

Because every model sits below the human effect in Chinese, a rise in English is a move towards human value rather than away from it. That bears on an explanation the origin result in Section 3.3 could only address indirectly. If the shortfall arose from models trained mainly on Western text being asked to judge a Chinese benchmark, then translating the materials into English should carry them further from that benchmark, not closer. Five of the six went the other way. Neither result rules out a role for corpus-level cultural mismatch. What they weaken together is the simpler account on which the small role effect follows from Western-trained models being asked to judge Chinese materials.

### 3.6 Naming an identity raised the role effect without closing the gap

Under zero-shot prompting, the model is given the scenario and the four questions and nothing else, so it is never told who it is. If the small role effect were merely a matter of never having been asked to answer as anyone in particular, supplying an identity should raise it. The persona prompt supplies one sentence as a system message: that the model is a Chinese university student answering according to its own impressions. The whole procedure was repeated with that sentence in place, all 12 models, Chinese materials, everything else unchanged, and the same models were fitted to the two sets of runs together.

It did move the role effect on gratitude by between −0.035 (Gemini-3.5-Flash-Lite) and 0.210 (DeepSeek-Pro), with 10 of 12 models showing an increase and the direction consistent across providers and tiers (Figure 5B; Supplementary Table S9). As in Section 3.5, these fits omit the by-scenario random slope for role, so the intervals are narrower than the design supports, and the counts are given for orientation rather than as claims.

It did not bring that effect to the human level. Under the persona prompt, it ran from 0.133 (Qwen3.7-Flash) to 0.807 (Doubao-2.1-Pro) against the human score of 0.756, with 11 of the 12 models still below the human estimate and three remaining entirely within the region of practical equivalence (Figure 5C). The gain is small relative to the distance it would have to cover: averaged over the 12 models, it is 0.10, compared to a mean shortfall of 0.44 under zero-shot prompting. It is also a generous version of the manipulation, supplying both an identity and an instruction to answer in the first person, so a smaller prompt would not be expected to yield more.

### 3.7 Robustness checks leave the results unchanged

First, we asked whether the role effect holds when the scenarios are split based on whether reversing the role also changes who the other person is. The split matters because the 24 scenarios are not alike in this respect. In 16 of them, the other person is a peer, a classmate, a roommate, or a stranger in a lift, and reversing the role leaves that person unchanged. In the remaining 8, it cannot: a student does not supervise their own advisor, so reversing the role replaces the advisor with a junior student, and the counterpart’s standing shifts with the role. In the 16 peer scenarios, the human role effect was 0.784 (95% HDI [0.451, 1.109]), slightly larger than the 0.756 estimated over all 24; in the 8 status-gap scenarios, it was 0.676 (95% HDI [0.311, 1.010]) and correspondingly less precise (Figure 6; Supplementary Table S9). The difference between the two was 0.108 (95% HDI [−0.118, 0.314], *pd* = 0.845). On the model side, 10 of the 12 placed that difference below zero, ranging from −0.519 (Qwen3.7-Max) to 0.203 (Claude-Opus-4.5), but no interval excluded zero. The effect is therefore not merely the change of counterpart under another name: it is present, and if anything larger, in the scenarios where only the perspective changes. These fits reported a small number of post-warmup transitions exceeding the maximum tree depth, which concerns sampling efficiency rather than the validity of the posterior; no model reported here had divergent transitions.

**Figure 6:**
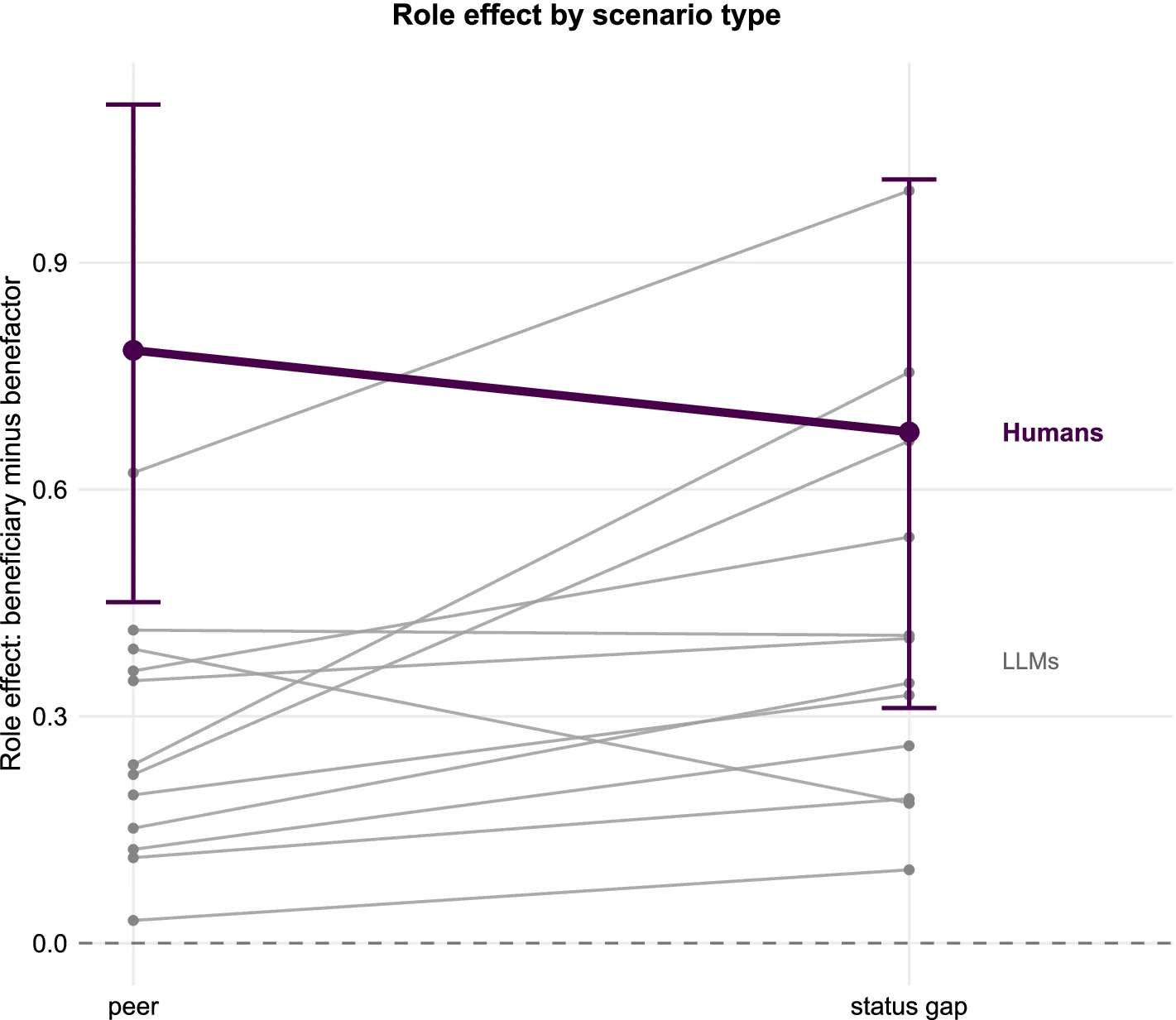
The role effect on gratitude in the 16 scenarios where the two parties are equals, compared to the 8 where reversing the role also reverses their standing. Purple represents the human data with its 95% highest density interval; grey lines are the 12 models, shown without intervals. Each system was fitted separately, so the human and model lines are estimates placed side by side rather than terms in one interaction (Values are given in Supplementary Table S9).

Second, we asked whether treating a five-point rating as a continuous quantity changes the picture. The scale is bounded and the ratings accumulate at both ends, so the Gaussian likelihood is an approximation; three models spanning the range of Figure 2B were refitted with a cumulative probit likelihood, which treats the five options as ordered categories and assumes nothing about the spacing between them. In latent standard deviation units, the role × agent term was −0.111 (95% HDI [−0.496, 0.283]) for Doubao-2.1-Pro, −0.527 (95% HDI [−0.944, −0.136]) for GPT-5.4, and −1.046 (95% HDI [−1.458, −0.685]) for DeepSeek-Flash. The units are not comparable to the scale points reported elsewhere, so what carries over is the sign and the ordering, and both are preserved: the same model is indistinguishable from the humans, and the same one is furthest from them. These refits also use a reduced random-effect structure, so the likelihood is not the only thing that differs between them and the main models. Treating the ratings as continuous therefore did not change the answer.

Third, we asked how much of the posterior comes from the data rather than from the prior, since every estimate here is the product of both, and a result that changes when the prior changes is a statement about the prior. We chose GPT-5.4 for this, since all 12 joint models share the same data volume, formula, and priors. We refitted it under a tighter and a wider slope prior, a fivefold change in scale, and the role × agent estimate went from −0.337 (95% HDI [−0.586, −0.089]) to −0.292 (95% HDI [−0.517, −0.059]) and −0.345 (95% HDI [−0.596, −0.088]). The three estimates span 0.053 scale points on intervals that almost entirely overlap. This estimate comes from the data, not from the prior.

Fourth, we recomputed the equivalence classification with the boundary drawn in three places rather than one. The boundary is half the human role effect on gratitude, and that effect is 0.758 when taken as the difference between the two groups’ raw means, giving ±0.379. Its posterior has a 95% interval of [0.422, 1.087], so half of it could as easily be 0.211 or 0.543. Supplementary Table S10 lists which models fall inside at each: DeepSeek-Flash alone at 0.211; Claude-Haiku-4.5, GPT-5.4-mini, and Qwen3.7-Flash as well at 0.379; and Claude-Opus-4.5, DeepSeek-Pro, and Gemini-3.5-Flash on top of those at 0.543, seven of 12. The 12 role effects are unchanged in all three cases, running from 0.047 to 0.727. The boundary therefore changes the count and nothing else: it sorts the 12 effects without altering them. The count is quoted three times, in the description of Figure 2B, in the tier comparison of Section 3.3, and in the persona result of Section 3.6, and in each case as a way of describing where the effects sit rather than as the quantity being compared. Sections 3.3 to 3.6 compare the effects themselves, so none of their conclusions depends on where the boundary is drawn.

## 4 Discussion

To assess whether language models reason about social situations the way people do, we compared 48 Chinese university students and 12 models, the flagship and the light model of three providers based in China and three based in English-speaking countries, on the same everyday helping scenarios. Our core manipulation was the perspective from which the episode is judged: that of the person giving help or that of the person receiving it, crossed with four situational versions. In the human baseline, the perspective mattered, most of all on gratitude, where the person reporting their own feelings gave a higher rating than the person estimating it for someone else. We found a dissociation in the models. They tracked how much help a situation called for as sharply as the humans did, and often more sharply. They separated the two perspectives much less, with only one model being indistinguishable from the humans on gratitude, and not on the other questions. What ordered the 12 was capability tier rather than the provider’s country, and neither writing the scenarios in English (the six models of the Chinese providers), nor stating an identity in the prompt, nor changing who the other person in the episode was brought the effect to the human level.

### 4.1 Judging the same help from two sides: the human asymmetry and where it comes from

On every one of the four questions, participants gave a higher rating when they answered as the person receiving help than as the person giving it, and on difficulty and gratitude, the interval excluded zero. Within one and the same episode, then, the person who performed the help rated it as having cost them less than the other side thought it had cost (2.32 against 2.86) and rated the other side as less grateful than that side reported being (3.41 against 4.16). This fits the account given by Flynn (2006). Reviewing work in organisational behaviour, social psychology, and sociology, Flynn (2006) argued that givers are obliged by a norm of generosity to downplay what they have done, while receivers are obliged by a norm of gratitude to emphasise it, and predicted that when both norms operate on one event, the two sides will report different magnitudes. Flynn (2006) named the pair the modesty bias, and it has since been used mainly as a frame rather than as something tested. Here, both of its sides were measured in the same participants and the same scenarios: a role effect of 0.540 on difficulty is a giver downplaying the cost, and one of 0.756 on gratitude is a giver underestimating what the help was worth.

We are not the first to test this prediction, but earlier tests measured how much people paid back rather than how they rated the episode. Zhang and Epley (2009), asking why what givers expect and what receivers give so often fail to match, varied the cost and the benefit of the same favour across six experiments and found that givers expected repayment scaled to the cost they had incurred, while receivers repaid in proportion to the benefit they had received. The asymmetry regarding cost held across their paradigms, but the one regarding benefit did not, and they traced both to what each side directly experiences. Flynn and Lake (2008) had participants make real requests of strangers on campus and predict beforehand how many people they would need to approach; the predictions ran to about twice the actual number. By separating the cost of the request from the cost of refusing it, they found that help seekers predicted based on what it costs to say yes, while whether people actually agreed depended on what it costs to say no. Zhao and Epley (2022) later confirmed the underestimate itself but not this account of it, so what carries over here is the finding rather than the mechanism. Flynn and Adams (2009) found that people who move between the two roles daily still cannot carry one across to the other: gift-givers expected a more expensive gift to be appreciated more, while recipients’ appreciation had nothing to do with price. All three studies point to the same conclusion. The side from which you respond determines what you have direct access to, and what you have access to influences the number you provide.

The difficulty result also explains an anomaly left by Tesser et al. (1968). They wrote three story themes: an aunt giving the reader a picture, a classmate helping build lab apparatus, and a neighbour helping paint the reader’s living room. They set the benefactor’s intention, the benefactor’s cost, and the value of the benefit at three levels each, resulting in 27 versions per theme. One hundred and twenty-six male undergraduates each read one version of each theme, answering as the recipient, and rated how grateful and how indebted they felt; the two correlated highly and were combined. All three factors had significant main effects, and none interacted, with regressions fitted on 60 cases per theme cross-validated on the rest at 0.73, 0.82, and 0.71. Cost, however, dropped out in the third theme. The three cost levels in that theme differed significantly from one another, and a paired-comparison study on a further sample confirmed that the scale anchors had not shifted, so neither explanation was available. The authors suggested that cost was the less stable predictor because, unlike the benefit to oneself and the benefactor’s intention towards oneself, it is not about the respondent. The present design makes that property something to manipulate. Of the four questions, difficulty is the only one about an externally observable cost rather than an internal state, so it is the one most exposed to whether the respondent bore that cost or watched someone else bear it. Its role effect, 0.540, was second only to gratitude. What Tesser et al. (1968) recorded as an unstable predictor can instead be read as cost moving with the position from which it is judged.

Position is not the only thing it moves with: Anicich et al. (2022) found the judged cost of a favour shifting with the recipient’s standing relative to the giver, even where the favour itself was held constant. The 0.540 recorded here is the part that moves with which side is answering, with standing held constant in 16 of the 24 scenarios and not differing between the two sets (Section 3.7).

Flynn (2006) made another prediction about perceived urgency that the present design also tests. The more pressing a recipient’s need, he argued, the more grateful they will feel and the more willing they will be to repay; but a helper who sees the need as urgent treats the help as appropriate rather than exceptional and expects less in return. If so, the two sides should diverge most when the need is not pressing. They do. Computed within each situation, the role effect on gratitude was largest under Unneeded, 0.973, and smallest under Pressing, 0.540. The cell means show why: under Unneeded, the benefactor side drops to 2.41 while the beneficiary side drops only to 3.38. The benefactor rates by whether the help was called for, while the beneficiary rates by the act itself, so when the need is obvious the two agree, and when there is no need they part ways. Flynn (2006) made this prediction without testing it; here it has a number.

The second contrast, how much the helper put in, cannot settle the question we set. Existing designs provided three directions, each from one side alone: gratitude was higher for requested help in Lee et al. (2019), marginally higher for offered help in Peng et al. (2018) and showed, no difference in Shang et al. (2021). Here, help that went beyond the minimum drew more gratitude than help that merely met it, and raised the helper’s apparent willingness and the difficulty attributed to them while leaving the recipient’s need untouched. We cannot adjudicate between the three findings above because our Considerate version raises what the helper put in as well as who raised the matter, and that confounding may be one source of the disagreement between them. Settling it would require a set of scenarios in which initiation alone varies. What our contrast does show is where the effect lands, on the two questions about the helper rather than on the recipient’s need, which is what a manipulation of the helper’s investment should do. That interpretation is closest to Peng et al. (2018). It is the smaller of our two situational contrasts, 0.232 against 1.325 for need, which is what should be expected when intention is present either way: the helper in the Baseline condition also intends to help, and only the degree of investment differs. When intention itself is removed, the effect is large, as when Yu et al. (2017) had a computer rather than a partner decide to bear part of a participant’s pain.

### 4.2 Only one model matched the human effect, and it matched only its size

Can language models reproduce the sensitivity human raters show to situational cues and to the side they answer from? The two have different answers. On situational cues, they did, and often more sharply than people: the contrast between a pressing need and one already met was 1.325 scale points in the humans, and 11 of the 12 were at or above it, 10 of them credibly above and the twelfth credibly below. On the side they answer from, 11 of the 12 fell credibly below the humans. The one exception is Doubao-2.1-Pro, and only on gratitude, where it was indistinguishable from the humans; on willingness and difficulty, it was credibly below them. Matching on gratitude alone is not matching the human role effect because the humans showed a credible role effect on two questions, difficulty and gratitude, and those two describe one and the same bias: the giver rates what the help cost him below what the beneficiary rates it, and rates the beneficiary’s gratitude below what the beneficiary reports. Difficulty and gratitude are also the two questions on which the models fell furthest short.

The models also tracked when the two sides should disagree most but still gave too little of it in every instance. The human role effect was largest under Unneeded and smallest under Pressing, and the models traced that shape; however, in all four situations, the human estimate sat above every model estimate. Cui et al. (2025) re-ran 156 scenario experiments from five management and psychology journals with three models and found main effects replicating 72.7 to 80.6% of the time, interactions only 45.7 to 62.9%, and effect sizes larger than the human ones. The present results show the same split and locate it: the models tracked the situation correctly in every condition but provided too little of it in every condition, and what they provided too little of is what changes depending on which side you are on.

### 4.3 The shortfall is not an artefact of how the task was presented

Could the small role effect in the models stem from how we presented the task? We ran two checks. Language: the 192 texts were translated into English and presented again to the six models of the Chinese providers. Five of the six showed a larger role effect, with intervals entirely above zero, while the situational contrasts in the same fits did not change, so the gain was specific to role. It still fell well below the human value. Identity: the zero-shot prompt never informs the model who it is, so we added one sentence stating that it is a Chinese university student answering according to its own impressions, and re-ran all 12. Ten increased, the largest gain being 0.210 against a human effect of 0.756, and 11 of the 12 still sat below the humans. Both checks raised the effect slightly, and neither brought it to the human level. The models were not failing to register which side they had been assigned; they registered it more accurately when it was marked more clearly, yet their answers did not separate accordingly.

The same disconnect has been located more precisely elsewhere. Ferraz et al. (2026) assigned five graded levels of a dark personality trait to 17 models playing the Ultimatum Game and collected over 200,000 decisions in each of the two roles. As proposers, where the model decides how to divide the money, fair offers fell from 91% at the lowest level to 17% at the highest, tracking the human gradient but at a third steeper. As responders, where the choice is between accepting €8 and taking nothing, acceptance stayed near 80% and barely moved with the trait at all. What makes the result useful is that they also read the justifications the models wrote alongside each choice: fairness words fell from 89 to 9% across the five levels and self-interest words rose from 4 to 72% in both roles. The language tracked the prompt completely while the behaviour did not. Their conclusion is that the disconnect is not between the prompt and the model’s processing but between that processing and what the model actually does. Nie et al. (2023) report a coarser version of the same phenomenon across tasks: injecting 25 personas into models answering 206 causal and moral judgement stories, agreement on moral judgements differed by 18.6% between the best and worst persona while causal judgements barely moved.

This also calls for one qualification to an account offered by Strachan et al. (2024). Comparing three models against human participants across 15 independent sessions on a battery of theory of mind measures, they found GPT-4 above human levels on hinting, irony, and strange stories, falling short only on faux pas. They propose in their discussion that the difficulty of the false belief task for humans lies in reconciling mental states that conflict with one’s own perspective, and that a system with no perspective of its own, because it is not driving a body through an environment, faces no comparable challenge. That account explains why models track another’s beliefs so readily, but it predicts something further: with no standpoint of one’s own to suppress, being assigned one should present no difficulty either. Our results do not bear out the second half. Having no perspective of one’s own makes tracking another’s states easy without making it easy to occupy one side, because occupying a side calls not for suppressing a standpoint but for having one.

The split also appears in a task with no role manipulation at all. Trott et al. (2026) ran 41 open-weight models on a false belief task with two manipulations. One was the knowledge state of the character, which determines whether the belief is true or false; only 14 of the 41 models were sensitive to it, none accounted statistically for the human effect, and the human effect size was above the entire distribution of model effect sizes. The other was the verb used to probe the belief, “John thinks the book is in the...” against “John looks for the book in the...”; here, the human effect size fell at the 49th percentile of the model distribution. Their interpretation is that the bias carried by a non-factive verb is lexical, learnable from how often “thinks” precedes something false, whereas tracking a knowledge state requires constructing a model of the situation. Two unrelated tasks, then, split the same way: what is written into the language is reproduced, and what has to be constructed is not. Wang et al. (2025) locate the reason in training. Scraped text rarely ties what was written to who wrote it, and likelihood losses reward the more probable output, so a model prompted with an identity should both misrepresent that group and flatten it, which they confirm across four models and 3,200 human participants. The asymmetry between the two sides leaves no trace in a corpus for the same reason: it is not a property of a helping episode but the difference between two readings of one, and a corpus records that episode once, from whichever side happened to write it down.

### 4.4 Capability tier ordered the 12; the provider’s country did not

We first asked whether the provider’s country mattered. If the shortfall came from a predominantly Western training corpus, the six models from Chinese providers should have performed better as a group, since the scenarios were written in Chinese and set on a Chinese campus. They did not. Both ends of the range are Chinese models, Doubao-2.1-Pro at the top and DeepSeek-Flash at the bottom. We then asked whether capability tier mattered, and here there was something: the six flagship models separated the two sides roughly twice as far as the six lighter ones did.

Although flagship models scored higher, we cannot claim they are stronger on this task: the interval includes zero, which, by our criterion throughout and by the frequentist standard of significance, means the difference is not established. Our result resonates with an unsettled literature. Two studies point in the same direction as we do. Trott et al. (2026) found parameter count to be the only significant predictor of false belief performance across 41 models, though no model reached the human baseline; thus, an ordering and a shortfall appeared together there as here. Hu et al. (2026) found simulation fidelity rising log-linearly with parameter count across 45 models, which they interpret as diminishing returns from scale. One study points in the opposite direction: Ferraz et al. (2026) found two frontier models behaving more extremely and less like humans than the open-source models in the Ultimatum Game. A fourth study suggests scale may not be the relevant variable at all. Pei et al. (2025) fingerprinted 18 models and found core reasoning converging among the top systems while alignment-related behaviours diverged sharply, which they attribute to developer alignment strategy rather than scale. Our flagship group is uneven in exactly that way, ranging from 0.727 for Doubao-2.1-Pro to 0.213 for Gemini-3.5-Flash, a spread wider than the gap between the two tiers. Our result therefore neither supports nor refutes the claim that capability brings a model closer to people, and what it does suggest is that tier alone does not account for the effect.

### 4.5 Limitations

First, all 24 scenarios were human-generated, with no involvement of language models, as they were written prior to the rise of LLMs. This has one advantage: the materials could not have been written to suit models as respondents. It also has a cost, as none of the tools that would now allow a close check of the wording were available at the time. Our four conditions, Baseline, Considerate, Pressing, and Unneeded, may not correspond to their names equally well in every text, and we cannot guarantee that the strength and manner of a manipulation are the same across scenarios. Specifically, 13 of the Considerate versions realise the condition by making the help more thorough or more costly, so that contrast varies with initiation together with the helper’s investment; in two scenarios, the Unneeded version withholds the helper’s timeliness or willingness rather than the recipient’s need, which means the need contrast reported here is, if anything, an overestimate. The need contrast and the role effect, which carry the results, are unaffected. We would encourage future designs of this kind to have several AI tools review the scenarios before the materials are finalised, checking version by version that each one alters only what it is meant to alter, and then to validate them with human raters. Our use of Bayesian mixed models addresses this internal variation: the by-scenario intercept and slope absorb it as noise unless it runs one way systematically. The situational contrasts are consistent with the manipulations having landed, but they are treatment effects on the rating dimensions themselves, not the independent checks we did not collect.

Second, the study has a single cultural setting: the materials are written in Chinese and the participants are all Chinese university students. Bond et al. (1982) note that self-effacement is normatively rewarded in this setting, so a benefactor’s downplaying of their own contribution may draw partly on that norm. That givers and receivers see the same act of help differently has also been reported in Western samples (Flynn and Lake, 2008; Zhang and Epley, 2009), so the asymmetry itself is not specific to China; however, those studies measured different quantities, so how large our 0.756 would be against a Western baseline remains unknown. Since we compare the models against this baseline, a shift in the baseline shifts the judgement of how far short they fall.

We would also emphasise that this study is not an assessment of model capability. We ask only whether a model’s ratings change with the side it is answering from, on this one set of materials. This neither measures general ability nor constitutes a comparison between providers. The association between capability tier and the role effect in Section 3.3 is a ranking rather than an established difference, and its interval includes zero; the provider’s country does not order the effect at all. Reading these results as a verdict on which models, or which companies, are better goes beyond what the design can support.

Third, each scenario describes one isolated act of help, with no prior exchange between the two parties, and all of them are campus-scale favours. Ding et al. (2025) show that gratitude tracks the prediction error between expected and received help, so that repeated help raises expectations and lowers the gratitude a given act elicits. Our one-shot vignettes still revealed a difference between the two sides, but we cannot say how that asymmetry behaves once expectations have had time to form, and we cannot speak to more consequential episodes.

Fourth, role was manipulated between participants: each person read only one version. The two groups of 24 differed as individuals, and any stable aspect of that difference would resemble a role effect. The trait measures show no difference on any of the 14 indices, the largest being neuroticism at *d* = −0.45 (Supplementary Table S11), but with 24 per group, the smallest detectable difference is *d* = 0.83, so they capture only gross imbalances. Group equivalence here is assumed by random assignment rather than demonstrated. The clearer cost is precision: the human effect on gratitude is 0.756 with a 95% interval of [0.422, 1.087], and the equivalence bound is drawn from that interval, which is why we recompute the classification at both ends (Section 3.7). Even so, 11 of the 12 models fall below 0.422, the lowest value that interval allows for people.

Fifth, the 12 models represent a snapshot. Providers update a model without changing its name, so the identifiers we report reflect what was queried but not what the same name will return later.

## 5 Conclusion

Machine psychology has drawn increasing attention as language models come into wider use (Hagendorff et al., 2023). We asked whether models judge the same helping episode differently depending on which side they answer from. On the surface, they largely can, tracking how much help a situation calls for as accurately as people do and often more sharply. What they fall short on is the social reasoning that depends on occupying a position: 11 of the 12 separated the two sides credibly less than humans, and the one exception matched on only one of the four questions. Our study suggests that work simulating human responses with these models should treat any quantity that depends on the respondent’s position as an underestimate. For everyday users, these models do understand a social situation. What they do not yet do, at least in the helping episodes we studied, is answer from a position within it. That is a small difference in scale points, but it is what makes a human response human, and it is worth knowing about in an era when these models increasingly answer on our behalf.

## Data Availability

The datasets for this study and the 24 scenarios can be found in the Open Science Framework at https://osf.io/76bcz/overview?view_only=34add04957d24bfbb8bbde71ca586c2b.
