# Supplementary material for: Can large language models reproduce humans’ perspective-dependent judgements of gratitude? Evidence from Claude, GPT, Gemini, DeepSeek, Qwen, and Doubao
Source: Front Psychol. 2026 Oct 14;17:1831256. doi: 10.3389/fpsyg.2026.1831256 (PMC13713510; doi:10.3389/fpsyg.2026.1831256)
Supplement: Supplementary file 1 [file Data_Sheet_1.PDF]

Can large language models reproduce humans' perspective-dependent judgments of gratitude? Evidence from Claude, GPT, Gemini, DeepSeek, Qwen, and Doubao

Supplementary Tables

Table S1. Model formulas by analysis stage

Model	Formula
Stage 1. Human baseline: random-effect structure	
h1	rating ~ condition_c * role_c + (1 | pid)
h2	rating ~ condition_c * role_c + (1 | pid) + (1 | scenario_id)
h3	rating ~ condition_c * role_c + (1 | pid) + (1 | scenario_id) + (1 | item)
h4	rating ~ condition_c * role_c + (1 | pid) + (1 + role_c | scenario_id) + (1 | item)
Stage 2. Human-model comparison: random-effect structure	
c1	rating ~ condition_c * role_c * agent_c + (1 | pid) + (1 | scenario_id)
c2	rating ~ condition_c * role_c * agent_c + (1 | pid) + (1 | scenario_id) + (1 | item)
c3	rating ~ condition_c * role_c * agent_c + (1 | pid) + (1 + role_c | scenario_id) + (1 | item)
Stage 3. Human against each model	
cmp	rating ~ condition_c * role_c * agent_c + (1 | pid) + (1 + role_c | scenario_id) + (1 | item)
Stage 4. Moderators: meta-regression on the twelve estimates	
m1	estimate | se(se) ~ 1 + (1 | obs)
m2	estimate | se(se) ~ tier + (1 | obs)
m3	estimate | se(se) ~ origin + (1 | obs)
m4	estimate | se(se) ~ tier + origin + (1 | obs)
Stage 5. Manipulations	
persona	rating ~ condition_c * role_c * persona + (1 | pid) + (1 | scenario_id)

Model	Formula
language	rating ~ condition_c * role_c * language + (1 | pid) + (1 | scenario_id)
symmetry	rating ~ condition_c * role_c * symmetry + (1 | pid) + (1 + role_c | scenario_id) + (1 | item)
Stage 6. Robustness	
ordinal	rating_ord ~ condition_c * role_c * agent_c + (1 | pid) + (1 | scenario_id)
prior	rating ~ condition_c * role_c * agent_c + (1 | pid) + (1 + role_c | scenario_id) + (1 | item)

Note. Formulas are read from the fitted objects, so the table cannot fall out of step with what was run. pid identifies the unit of analysis: a participant on the human side, one independent run of a model on the other. role and agent carry Helmert contrasts and condition sum-to-zero coding, so that each lower-order term is an average over the levels of the others. All models are Gaussian except the ordinal robustness check, which is cumulative probit. Priors: Normal(0, 1) on slopes, Normal(3, 1) on the intercept, Exponential(1) on standard deviations. Sampling: 3000 iterations with 1500 warmup on 8 chains.

Table S2. Model comparison by leave-one-out cross-validation

Model	LOOIC	Δ	Weight (%)	R2
Human baseline				
h_model_4	10742.2	0.0	100.0	0.441 [0.049, 0.597]
h_model_3	10768.6	26.5	0.0	0.438 [0.049, 0.594]
h_model_2	10969.3	227.2	0.0	0.419 [0.047, 0.569]
h_model_1	11329.1	587.0	0.0	0.391 [0.044, 0.532]
Human-model comparison				
c_model_3	20590.7	0.0	100.0	0.513 [0.054, 0.689]
c_model_2	20933.6	342.9	0.0	0.505 [0.053, 0.678]
c_model_1	22434.8	1844.1	0.0	0.464 [0.049, 0.624]

Note. Lower LOOIC indicates better expected predictive accuracy. Δ is the difference from the best model in each stage; weight is the Akaike weight computed over the models within a stage. LOO is preferred to WAIC throughout because individual observations are influential enough for the WAIC diagnostic to flag instability. R^2 is the Bayesian R^2 with its 95% credible interval.

Table S3. The human effects: role and situation on each rating dimension

Dimension	Estimate	HDI	pd	C1 concern	C2 urgency
willingness	0.243	[-0.054, 0.538]	.946	0.402 [0.295, 0.527]	0.228 [0.118, 0.333]
need	0.259	[-0.013, 0.546]	.964	-0.068 [-0.179, 0.037]	1.961 [1.862, 2.067]
difficulty	0.540	[0.160, 0.908]	.998	0.422 [0.331, 0.518]	0.216 [0.124, 0.306]
gratitude	0.756	[0.422, 1.087]	> .999	0.232 [0.147, 0.322]	1.325 [1.241, 1.408]

Note. The role effect is the beneficiary minus benefactor difference in scale points, averaged over the four situations. C1 contrasts a helper who shows extra care against the unmarked baseline; C2 contrasts a pressing need against one already met. Estimates are posterior means with 95% highest density intervals; pd is the probability of direction. n = 48 participants, 18,432 ratings.

Table S4. Human cell means by dimension, situation and role

Situation	Beneficiary	Benefactor
difficulty		
Baseline	2.71 [2.38, 3.03]	2.25 [1.94, 2.58]
Considerate	3.21 [2.89, 3.53]	2.59 [2.26, 2.90]
Pressing	2.88 [2.56, 3.20]	2.31 [2.00, 2.63]
Unneeded	2.63 [2.32, 2.96]	2.13 [1.81, 2.45]
gratitude		
Baseline	4.21 [3.95, 4.48]	3.58 [3.32, 3.85]
Considerate	4.56 [4.30, 4.82]	3.69 [3.43, 3.96]
Pressing	4.49 [4.24, 4.77]	3.95 [3.68, 4.21]
Unneeded	3.38 [3.13, 3.66]	2.41 [2.14, 2.67]
need		
Baseline	4.04 [3.75, 4.34]	3.90 [3.61, 4.18]
Considerate	4.08 [3.79, 4.38]	3.72 [3.44, 4.00]
Pressing	4.49 [4.18, 4.78]	4.34 [4.07, 4.63]
Unneeded	2.64 [2.34, 2.94]	2.26 [1.99, 2.55]
willingness		
Baseline	3.98 [3.72, 4.24]	3.85 [3.59, 4.10]
Considerate	4.53 [4.27, 4.78]	4.10 [3.85, 4.36]
Pressing	3.82 [3.57, 4.09]	3.96 [3.70, 4.21]
Unneeded	3.94 [3.69, 4.21]	3.38 [3.14, 3.65]

Note. Posterior means with 95% highest density intervals on the 1-5 rating scale, from the model fitted to each dimension separately, averaged over the 24 scenarios. Role was manipulated between participants, so the two columns come from different groups of 24 and the difference between them is a between-group comparison. The role effects averaged over the four situations are in the preceding table.

Table S5. Role effect within each model, by rating dimension

Model	willingness	need	difficulty	gratitude
Doubao-2.1-Pro	-0.142 [-0.305, 0.020]	0.354 [0.025, 0.662]	0.210 [0.054, 0.372]	0.727 [0.516, 0.939]
GPT-5.4	-0.101 [-0.263, 0.064]	0.088 [-0.140, 0.311]	0.162 [0.016, 0.310]	0.403 [0.231, 0.567]
Gemini-3.5-Flash-Lite	0.120 [-0.097, 0.314]	0.209 [-0.118, 0.519]	0.183 [-0.019, 0.389]	0.401 [0.152, 0.640]
Qwen3.7-Max	-0.066 [-0.241, 0.111]	0.377 [0.036, 0.694]	0.108 [-0.035, 0.255]	0.394 [0.164, 0.631]
DeepSeek-Pro	-0.040 [-0.198, 0.122]	0.034 [-0.155, 0.236]	0.115 [-0.039, 0.267]	0.359 [0.222, 0.496]
Doubao-2.1-Turbo	-0.148 [-0.317, 0.022]	0.166 [-0.103, 0.413]	0.247 [0.135, 0.354]	0.357 [0.162, 0.551]
Claude-Opus-4.5	0.059 [-0.100, 0.209]	-0.088 [-0.290, 0.111]	0.114 [-0.045, 0.287]	0.316 [0.173, 0.472]
GPT-5.4-mini	0.042 [-0.164, 0.252]	0.068 [-0.102, 0.245]	0.124 [-0.030, 0.272]	0.231 [0.103, 0.360]
Gemini-3.5-Flash	-0.177 [-0.386, 0.013]	0.094 [-0.105, 0.298]	0.166 [-0.017, 0.340]	0.213 [0.044, 0.381]
Claude-Haiku-4.5	-0.131 [-0.303, 0.039]	0.068 [-0.234, 0.359]	0.092 [-0.070, 0.256]	0.144 [-0.029, 0.295]
Qwen3.7-Flash	-0.123 [-0.259, 0.019]	0.289 [0.089, 0.477]	0.068 [-0.084, 0.211]	0.138 [0.044, 0.232]
DeepSeek-Flash	-0.008 [-0.190, 0.166]	0.023 [-0.133, 0.187]	0.091 [-0.041, 0.224]	0.047 [-0.070, 0.173]

Note. Posterior means with 95% highest density intervals, in scale points, averaged over the four situations. Positive values indicate the pattern people show: a higher rating when judging as the person receiving help. For comparison the human effects are willingness 0.243, need 0.259, difficulty 0.540, gratitude 0.756. Ordered by the gratitude estimate.

Table S6. Departure from the human role effect, by dimension

Model	willingness	need	difficulty	gratitude
Claude-Haiku-4.5	-0.370 [-0.568, -0.169]	-0.181 [-0.384, 0.019]	-0.452 [-0.710, -0.176]	-0.596 [-0.834, -0.339]
Claude-Opus-4.5	-0.177 [-0.388, 0.024]	-0.342 [-0.549, -0.128]	-0.430 [-0.701, -0.172]	-0.420 [-0.672, -0.187]
DeepSeek-Flash	-0.244 [-0.449, -0.038]	-0.225 [-0.432, -0.017]	-0.451 [-0.712, -0.180]	-0.685 [-0.940, -0.446]
DeepSeek-Pro	-0.279 [-0.489, -0.066]	-0.214 [-0.415, -0.008]	-0.438 [-0.694, -0.173]	-0.389 [-0.626, -0.147]
Doubao-2.1-Pro	-0.374 [-0.588, -0.163]	0.102 [-0.106, 0.308]	-0.339 [-0.601, -0.067]	-0.022 [-0.267, 0.224]
Doubao-2.1-Turbo	-0.385 [-0.595, -0.180]	-0.089 [-0.285, 0.128]	-0.304 [-0.577, -0.035]	-0.376 [-0.613, -0.125]
GPT-5.4	-0.337 [-0.546, -0.132]	-0.160 [-0.369, 0.043]	-0.385 [-0.652, -0.122]	-0.337 [-0.586, -0.089]
GPT-5.4-mini	-0.200 [-0.405, 0.016]	-0.180 [-0.387, 0.032]	-0.425 [-0.692, -0.146]	-0.506 [-0.768, -0.266]
Gemini-3.5-Flash	-0.409 [-0.617, -0.204]	-0.157 [-0.361, 0.046]	-0.384 [-0.644, -0.114]	-0.519 [-0.765, -0.269]
Gemini-3.5-Flash-Lite	-0.116 [-0.322, 0.085]	-0.044 [-0.243, 0.172]	-0.361 [-0.632, -0.112]	-0.337 [-0.589, -0.087]
Qwen3.7-Flash	-0.361 [-0.579, -0.150]	0.039 [-0.162, 0.253]	-0.480 [-0.746, -0.208]	-0.604 [-0.840, -0.355]
Qwen3.7-Max	-0.301 [-0.512, -0.093]	0.131 [-0.079, 0.326]	-0.439 [-0.700, -0.174]	-0.342 [-0.589, -0.096]

Note. Each cell is the difference of differences: the model's role effect minus the human role effect on that dimension, in scale points. Negative values indicate a smaller asymmetry than people show. No region of practical equivalence is reported: the bound used elsewhere is half the human role effect, which answers whether a system shows the asymmetry at all, and zero on this scale means indistinguishable from people rather than absent.

Table S7. Situational effects and their departure from the human ones, gratitude

Model	Need (own)	Care (own)	Need × agent	Care × agent
Claude-Haiku-4.5	1.370 [1.304, 1.435]	0.513 [0.445, 0.585]	0.183 [0.114, 0.249]	0.277 [0.211, 0.344]
Claude-Opus-4.5	1.371 [1.307, 1.432]	0.607 [0.540, 0.671]	0.042 [-0.023, 0.106]	0.284 [0.217, 0.347]
DeepSeek-Flash	1.529 [1.464, 1.597]	0.249 [0.180, 0.320]	0.229 [0.161, 0.303]	0.057 [-0.015, 0.128]
DeepSeek-Pro	1.634 [1.562, 1.703]	0.406 [0.332, 0.476]	0.371 [0.297, 0.445]	0.243 [0.168, 0.312]
Doubao-2.1-Pro	1.388 [1.314, 1.458]	0.291 [0.213, 0.366]	0.213 [0.137, 0.288]	0.113 [0.042, 0.193]
Doubao-2.1-Turbo	1.159 [1.090, 1.225]	0.337 [0.266, 0.410]	-0.071 [-0.145, -0.001]	0.118 [0.047, 0.188]
GPT-5.4	1.777 [1.711, 1.848]	0.367 [0.297, 0.436]	0.520 [0.449, 0.591]	0.156 [0.085, 0.227]
GPT-5.4-mini	1.541 [1.473, 1.609]	0.461 [0.391, 0.534]	0.256 [0.185, 0.328]	0.203 [0.128, 0.273]
Gemini-3.5-Flash	1.825 [1.757, 1.893]	0.309 [0.237, 0.381]	0.576 [0.508, 0.647]	0.161 [0.091, 0.229]
Gemini-3.5-Flash-Lite	1.662 [1.591, 1.736]	0.256 [0.183, 0.337]	0.482 [0.406, 0.552]	0.083 [0.008, 0.156]
Qwen3.7-Flash	1.535 [1.462, 1.612]	0.222 [0.139, 0.300]	0.178 [0.092, 0.264]	0.039 [-0.046, 0.125]
Qwen3.7-Max	1.609 [1.536, 1.683]	0.340 [0.262, 0.417]	0.392 [0.313, 0.467]	0.155 [0.077, 0.233]

Note. The first two columns are each model's own contrast; the last two are that contrast minus the human one, where a positive value indicates a sharper reading than people draw. Need contrasts a pressing need against one the help does not answer; care contrasts a helper whose care exceeds what the situation minimally asked for against the unmarked baseline. The human contrasts were 1.325 and 0.232 respectively. Read against the two preceding tables: the same models that read the situation at least as sharply as people read the role less sharply.

Table S8. What predicts the size of a model's role effect

Level	Estimate	HDI	pd
Between-model SD			
intercept only	0.159	[0.078, 0.265]	
+ tier	0.120	[0.027, 0.236]	
+ origin	0.169	[0.081, 0.286]	
+ tier + origin	0.131	[0.042, 0.256]	
Contrast			
flagship - light	0.186	[-0.023, 0.367]	.967
Western - Chinese	-0.040	[-0.241, 0.148]	.670
Group mean			
light	0.220		
flagship	0.402		
Chinese	0.337		
Western	0.285		

Note. A meta-regression on the twelve role effects reported above, each weighted by its standard error. Capability tier and provider origin are properties of a model rather than of a rating, so there are twelve observations of each; fitting them on the ratings themselves would add no information about the moderators. Contrasts are in scale points and both include zero, so the ordering in the figures carries more of the pattern than these estimates do. Between-model SD is the spread of true role effects once each estimate's own measurement error is removed; a moderator that matters shrinks it. Leave-one-out is not estimable for this model class, so no information criteria are reported. n = 12 models.

Table S9. Three manipulations of the role effect

Model	Estimate	HDI	pd
English against Chinese materials			
DeepSeek-Flash	0.050	[0.005, 0.094]	.985
DeepSeek-Pro	0.180	[0.129, 0.227]	> .999
Doubao-2.1-Pro	0.129	[0.077, 0.179]	> .999
Doubao-2.1-Turbo	0.224	[0.181, 0.267]	> .999
Qwen3.7-Max	0.088	[0.038, 0.142]	> .999
Qwen3.7-Flash	-0.155	[-0.217, -0.092]	> .999
Peer against status-gap scenarios			
HUMAN	0.108	[-0.118, 0.314]	.845
Claude-Opus-4.5	0.203	[-0.291, 0.711]	.787
Claude-Haiku-4.5	-0.137	[-0.701, 0.409]	.705
DeepSeek-Flash	-0.067	[-0.470, 0.311]	.640
DeepSeek-Pro	-0.056	[-0.488, 0.378]	.606
Doubao-2.1-Pro	-0.373	[-1.049, 0.350]	.849
Doubao-2.1-Turbo	-0.441	[-1.068, 0.175]	.917
Gemini-3.5-Flash	-0.192	[-0.738, 0.331]	.764
Gemini-3.5-Flash-Lite	-0.177	[-0.917, 0.606]	.682
GPT-5.4	0.008	[-0.609, 0.632]	.504
GPT-5.4-mini	-0.131	[-0.597, 0.319]	.721
Qwen3.7-Max	-0.519	[-1.245, 0.206]	.921
Qwen3.7-Flash	-0.078	[-0.421, 0.247]	.687
Role effect in peer scenarios			
HUMAN	0.784	[0.451, 1.109]	> .999
Claude-Opus-4.5	0.389	[0.098, 0.695]	.994
Claude-Haiku-4.5	0.124	[-0.206, 0.452]	.772
DeepSeek-Flash	0.030	[-0.205, 0.242]	.603
DeepSeek-Pro	0.347	[0.101, 0.605]	.996
Doubao-2.1-Pro	0.622	[0.204, 1.074]	.996
Doubao-2.1-Turbo	0.223	[-0.153, 0.596]	.878
Gemini-3.5-Flash	0.152	[-0.160, 0.466]	.834
Gemini-3.5-Flash-Lite	0.360	[-0.092, 0.827]	.943
GPT-5.4	0.414	[0.046, 0.782]	.987
GPT-5.4-mini	0.196	[-0.072, 0.455]	.934
Qwen3.7-Max	0.236	[-0.195, 0.667]	.860
Qwen3.7-Flash	0.113	[-0.073, 0.300]	.891

Model	Estimate	HDI	pd
Role effect in status-gap scenarios			
HUMAN	0.676	[0.311, 1.010]	> .999
Claude-Opus-4.5	0.185	[-0.227, 0.610]	.817
Claude-Haiku-4.5	0.261	[-0.191, 0.725]	.876
DeepSeek-Flash	0.097	[-0.205, 0.426]	.728
DeepSeek-Pro	0.403	[0.057, 0.768]	.986
Doubao-2.1-Pro	0.995	[0.408, 1.582]	.999
Doubao-2.1-Turbo	0.664	[0.128, 1.179]	.991
Gemini-3.5-Flash	0.344	[-0.112, 0.789]	.937
Gemini-3.5-Flash-Lite	0.537	[-0.132, 1.164]	.947
GPT-5.4	0.407	[-0.116, 0.922]	.938
GPT-5.4-mini	0.328	[-0.059, 0.705]	.952
Qwen3.7-Max	0.755	[0.148, 1.371]	.989
Qwen3.7-Flash	0.191	[-0.087, 0.453]	.922
Role effect under the persona prompt			
Claude-Opus-4.5	0.416	[0.391, 0.441]	> .999
Claude-Haiku-4.5	0.328	[0.300, 0.356]	> .999
DeepSeek-Flash	0.152	[0.122, 0.183]	> .999
DeepSeek-Pro	0.572	[0.540, 0.606]	> .999
Doubao-2.1-Pro	0.807	[0.773, 0.842]	> .999
Doubao-2.1-Turbo	0.495	[0.465, 0.527]	> .999
Gemini-3.5-Flash	0.368	[0.337, 0.399]	> .999
Gemini-3.5-Flash-Lite	0.376	[0.342, 0.408]	> .999
GPT-5.4	0.457	[0.429, 0.488]	> .999
GPT-5.4-mini	0.375	[0.346, 0.403]	> .999
Qwen3.7-Max	0.512	[0.479, 0.548]	> .999
Qwen3.7-Flash	0.133	[0.089, 0.177]	> .999
Role effect under the zero-shot prompt			
Claude-Opus-4.5	0.317	[0.293, 0.343]	> .999
Claude-Haiku-4.5	0.145	[0.117, 0.173]	> .999
DeepSeek-Flash	0.049	[0.018, 0.078]	.999
DeepSeek-Pro	0.363	[0.331, 0.395]	> .999
Doubao-2.1-Pro	0.729	[0.694, 0.764]	> .999
Doubao-2.1-Turbo	0.362	[0.331, 0.392]	> .999
Gemini-3.5-Flash	0.215	[0.184, 0.246]	> .999
Gemini-3.5-Flash-Lite	0.411	[0.377, 0.443]	> .999

Model	Estimate	HDI	pd
GPT-5.4	0.407	[0.378, 0.436]	> .999
GPT-5.4-mini	0.235	[0.206, 0.264]	> .999
Qwen3.7-Max	0.397	[0.362, 0.432]	> .999
Qwen3.7-Flash	0.139	[0.094, 0.181]	> .999
Stating an identity			
Claude-Opus-4.5	0.098	[0.063, 0.132]	> .999
Claude-Haiku-4.5	0.183	[0.142, 0.221]	> .999
DeepSeek-Flash	0.102	[0.061, 0.145]	> .999
DeepSeek-Pro	0.210	[0.166, 0.257]	> .999
Doubao-2.1-Pro	0.078	[0.028, 0.126]	.999
Doubao-2.1-Turbo	0.133	[0.090, 0.173]	> .999
Gemini-3.5-Flash	0.152	[0.108, 0.197]	> .999
Gemini-3.5-Flash-Lite	-0.035	[-0.082, 0.011]	.930
GPT-5.4	0.050	[0.009, 0.090]	.993
GPT-5.4-mini	0.139	[0.099, 0.181]	> .999
Qwen3.7-Max	0.115	[0.067, 0.166]	> .999
Qwen3.7-Flash	-0.007	[-0.067, 0.054]	.581

Note. Each entry is a difference between two role effects, in scale points, on the gratitude dimension. English against Chinese: the same scenarios translated, 6 models run in both. Stating an identity: a system prompt naming the model as a Chinese university student, against no system prompt. Peer against status-gap: the 16 scenarios where the two parties are equals against the 8 where reversing the role also reverses their standing; the human entry is included here because that comparison is the one place a human value is directly informative about the models.

Table S10. Sensitivity of the equivalence classification to the bound

Basis	Bound	Models inside	Models
Lower end of the human 95% HDI	0.211	1 of 12	DeepSeek-Flash
Bound used in the main analysis	0.379	4 of 12	Claude-Haiku-4.5, DeepSeek-Flash, GPT-5.4-mini, Qwen3.7-Flash
Upper end of the human 95% HDI	0.543	7 of 12	Claude-Opus-4.5, Claude-Haiku-4.5, DeepSeek-Flash, DeepSeek-Pro, Gemini-3.5-Flash, GPT-5.4-mini, Qwen3.7-Flash

Note. The region of practical equivalence is half the human role effect, and that effect is estimated rather than known. Recomputing the classification with the bound built from the ends of the human 95% interval instead shows how far the reading depends on the choice: the count runs from 1 to 7 of 12. A model counts as inside only when its whole 95% interval lies within the bound. The classification is therefore reported throughout as sensitive to the bound rather than as a property of the models.

Table S11. Internal consistency and group comparison on the trait measures

**Measure**	*α*	**Beneficiary M (SD)**	**Benefactor M (SD)**	*t*	*df*	*p*	***d* [95% CI]**
**TAS-20**							
Difficulty identifying feelings	.83	2.17 (0.79)	2.10 (0.81)	0.31	46.0	.760	0.09 [−0.48, 0.65]
Difficulty describing feelings	.63	2.68 (0.87)	2.79 (0.75)	−0.46	45.1	.645	−0.13 [−0.70, 0.43]
Externally oriented thinking	.60	2.45 (0.62)	2.27 (0.64)	1.00	46.0	.321	0.29 [−0.28, 0.86]
Total	.79	2.41 (0.58)	2.34 (0.50)	0.45	45.2	.652	0.13 [−0.44, 0.70]
**IRI**							
Perspective taking	.61	3.62 (0.48)	3.68 (0.57)	−0.39	44.6	.699	−0.11 [−0.68, 0.45]
Fantasy	.80	3.46 (0.91)	3.33 (0.74)	0.52	44.2	.604	0.15 [−0.42, 0.72]
Empathic concern	.77	3.77 (0.66)	3.78 (0.68)	−0.03	46.0	.976	−0.01 [−0.57, 0.56]
Personal distress	.65	3.27 (0.60)	3.46 (0.60)	−1.13	46.0	.263	−0.33 [−0.90, 0.24]
Total	.85	3.53 (0.50)	3.57 (0.45)	−0.25	45.4	.804	−0.07 [−0.64, 0.49]
**LSAS**							
Avoidance	.88	30.79 (10.21)	33.17 (12.26)	−0.73	44.5	.470	−0.21 [−0.78, 0.36]
Fear	.93	22.04 (11.65)	22.92 (13.22)	−0.24	45.3	.809	−0.07 [−0.64, 0.50]
Total	—	52.83 (19.09)	56.08 (22.37)	−0.54	44.9	.591	−0.16 [−0.72, 0.41]
**BFI-2**							

Extraversion	.88	2.93 (0.64)	2.98 (0.90)	−0.20	41.6	.842	−0.06 [−0.62, 0.51]
Agreeableness	.77	3.59 (0.55)	3.72 (0.50)	−0.82	45.6	.414	−0.24 [−0.81, 0.33]
Conscientiousness	.88	3.38 (0.83)	3.52 (0.64)	−0.70	43.1	.488	−0.20 [−0.77, 0.37]
Neuroticism	.88	2.64 (0.60)	2.98 (0.88)	−1.57	40.6	.123	−0.45 [−1.03, 0.12]
Openness	.85	3.40 (0.70)	3.68 (0.76)	−1.32	45.8	.194	−0.38 [−0.95, 0.19]

Note. n = 24 per group. TAS-20 = Toronto Alexithymia Scale (Bagby et al., 1994); IRI = Interpersonal Reactivity Index (Davis, 1983); LSAS = Liebowitz Social Anxiety Scale (Heimberg et al., 1999); BFI-2 = Big Five Inventory-2 (Soto & John, 2017; Chinese version, Zhang et al., 2022). TAS-20, IRI and BFI-2 scores are item means on 1–5 scales; LSAS scores are sums on 0–3 items. Cronbach's α is computed on the present sample; the LSAS total is the sum of two separately administered parts, so no α is given. Group differences are Welch's t tests, beneficiary minus benefactor, with Cohen's d based on the pooled *SD*; Holm-corrected p values across the fourteen subscales were all 1.000. Totals are nested within subscales and the fifteen BFI-2 facets are available with the deposited data. These comparisons are descriptive and do not establish equivalence: with 24 per group the smallest detectable difference is d = 0.83, so they rule out a gross imbalance and nothing finer. Group equivalence rests on random assignment. The trait measures enter no model in the main analysis.
